# Identification of the X-linked germ cell specific miRNAs (XmiRs) and their functions

**DOI:** 10.1371/journal.pone.0211739

**Published:** 2019-02-01

**Authors:** Hiromitsu Ota, Yumi Ito-Matsuoka, Yasuhisa Matsui

**Affiliations:** 1 Cell Resource Center for Biomedical Research, Institute of Development, Aging and Cancer, Tohoku University, Sendai, Miyagi, Japan; 2 The Japan Agency for Medical Research and Development-Core Research for Evolutional Science and Technology (AMED-CREST), Chuo-ku, Tokyo, Japan; 3 Graduate School of Life Sciences, Tohoku University, Sendai, Miyagi, Japan; Centre de Recherche en Cancerologie de Lyon, FRANCE

## Abstract

MicroRNAs (miRNAs) play a critical role in multiple aspects of biology. Dicer, an RNase III endonuclease, is essential for the biogenesis of miRNAs, and the germ cell-specific *Dicer1* knockout mouse shows severe defects in gametogenesis. How miRNAs regulate germ cell development is still not fully understood. In this study, we identified germ cell-specific miRNAs (miR-741-3p, miR-871-3p, miR-880-3p) by analyzing published RNA-seq data of mouse. These miRNA genes are contiguously located on the X chromosome near other miRNA genes. We named them X chromosome-linked miRNAs (XmiRs). To elucidate the functions of XmiRs, we generated knockout mice of these miRNA genes using the CRISPR/Cas9-mediated genome editing system. Although no histological abnormalities were observed in testes of F0 mice in which each miRNA gene was disrupted, a deletion covering *miR-871* and *miR-880* or covering all *XmiRs* (*ΔXmiRs*) resulted in arrested spermatogenesis in meiosis in a few seminiferous tubules, indicating their redundant functions in spermatogenesis. Among candidate targets of XmiRs, we found increased expression of a gene encoding a WNT receptor, FZD4, in *ΔXmiRs* testis compared with that in wildtype testis. miR-871-3p and miR-880-3p repressed the expression of *Fzd4* via the 3′-untranslated region of its mRNA. In addition, downstream genes of the WNT/β-catenin pathway were upregulated in *ΔXmiRs* testis. We also found that *miR-871*, *miR-880*, and *Fzd4* were expressed in spermatogonia, spermatocytes and spermatids, and overexpression of *miR-871* and *miR-880* in germ stem cells in culture repressed their increase in number and *Fzd4* expression. Previous studies indicated that the WNT/β-catenin pathway enhances and represses proliferation and differentiation of spermatogonia, respectively, and our results consistently showed that stable β-catenin enhanced GSC number. In addition, stable β-catenin partially rescued reduced GSC number by overexpression of *miR-871* and *miR-880*. The results together suggest that *miR-871* and *miR-880* cooperatively regulate the WNT/β-catenin pathway during testicular germ cell development.

## Introduction

Germ cells first arise as primordial germ cells (PGCs) in early embryos [1], and they proliferate and migrate into the genital ridges during embryonic development [2]. After arriving at the genital ridges, male germ cells enter G0 mitotic arrest and differentiate into prospermatogonia, which resume proliferation after birth [3, 4]. Subsequently, they further differentiate into spermatogonial stem cells (SSCs), and a subpopulation of SSCs starts the first wave of spermatogenesis [5, 6]. Spermatogenesis is a highly complex differentiation process, during which gene expression is highly orchestrated and strictly regulated [7].

In addition to transcriptional regulation, post-transcriptional regulation also plays an important role during spermatogenesis. MicroRNAs (miRNAs) are small non-coding RNAs 18–23 nucleotides in length that play a critical role in the regulation of development and differentiation in many organisms and different tissues [8, 9]. For example, miR-125a modulates self-renewal of hematopoietic stem cells and protects them from apoptosis [10]. miR-1 and miR-206 inhibit cell proliferation and promote myoblast differentiation [11]. miR-29 has multiple activities at different stages of osteoblast differentiation [12]. miRNAs repress gene expression by binding the 3′-untranslated region (UTR) of their target mRNAs, thereby decreasing mRNA stability and translational efficiency [13].

*Dicer1* encodes an RNase III endonuclease, which is a key enzyme for the biogenesis of miRNAs. Germ cell-specific *Dicer1* knockout affects PGC proliferation, spermatogenesis, and fertility. For example, specific removal of *Dicer1* in male germ cells with *Ddx4-Cre* results in deficient transition from the leptotene to zygotene stage in meiosis, increased apoptosis in the pachytene stage, and morphological defects in spermatozoa [14, 15]. Germ cell-specific knockout mice of *Drosha*, which encodes an RNase III endonuclease that processes primary miRNAs to pre-miRNAs, with germ cell-specific *Stra8-Cre* also leads to progressive loss of pachytene spermatocytes and spermatids [16]. In addition, previous studies reported that various miRNAs were expressed in germ cells and are involved in their development [17]. For instance, survival and/or proliferation [18–22], and differentiation [23–25] of SSCs are controlled by several miRNAs via inhibiting their target gene expression. However, miRNAs-mediated regulation of complex spermatogenic processes have not been fully understood.

WNT/β-catenin signaling is a highly conserved pathway that is essential for embryonic development and cellular differentiation. For example, WNT5A stimulates the proliferation and self-renewal of hematopoietic stem cells *in vitro* [26], and WNT10B plays an important role in bone development [27]. In the WNT/β-catenin signaling pathway, WNTs bind to members of the Frizzled (FZD) family of receptors and form a stable ligand-receptor complex [28, 29]. This complex phosphorylates the intra-cellular Dishevelled protein and inhibits glycogen synthase kinase-3β, which induces ubiquitination and subsequent degradation of β-catenin via phosphorylation; thereby, WNTs reduce degradation of β-catenin [30]. Hence, cytoplasmic levels of β-catenin rise, and β-catenin translocates to the nucleus where it associates with T-cell factor/lymphoid enhancer binding factor transcription factors to activate the expression of downstream genes [31, 32]. WNT/β-catenin signaling stimulates proliferation of SSCs but represses their differentiation potential [33–35], and is also involved in later stages of spermatogenesis [36–38].

In this study, we identified germ cell-specific, X chromosome-linked miRNAs (XmiRs; miR-741-3p, miR-871-3p, miR-880-3p). We generated knockout mice of these miRNA genes. The phenotype of the mice suggested that miR-871-3p and miR-880-3p were functionally redundant and that their deficiency caused spermatogenic failure by abnormally activating the WNT/β-catenin signaling pathway. In vitro studies by using germ stem cells (GSCs) revealed that WNT/β-catenin signaling was under the control of miR-871-3p and miR-880-3p to regulate GSC number.

## Materials and methods

### Animals

MCH and B6D2F1 (C57BL/6 x DBA2 F1) were purchased from CLEA Japan Inc. and Japan SLC., respectively. Oct4-deltaPE-GFP [39] transgenic mice were maintained in a C57BL/6J genetic background. All animal care and experiments were carried out in according to the guidelines for experimental animals defined by the facility, the Animal Unit of the Institute of Development, Aging and Cancer (Tohoku University). Animal protocols were reviewed and approved by the Tohoku University Animal Studies Committee.

### miRNA sequence analysis

Small RNA sequence data using in this study are as follows [40–42]: GSE40499; Brain (SRR553582), Cerebellum (SRR553583), Heart (SRR553584), Kidney (SRR553585), Testes (SRR553586), GSE52950; ES cells (SRR1042095, SRR1042096, SRR1042097), MEF cells (SRR1042098, SRR1042099), GSE59254; PGC (SRR15097510), Spermatogonia (SRR1509750), Spermatozoa (SRR1509748). Cutadapt (Ver 1.8.3) was used to clip adapter sequences from raw small RNAs sequencing data. Prinseq (Ver. 0.20.4) was then used to filter low quality reads out from clipped reads. The processed reads were aligned to mouse-genomic reference (mm10) or miRBase 21 reference by using aligner program Bowtie (Ver 1.1.2). Heatmaps were built using the ‘regHeatmap’ function of the ‘Heatplus’ package of R.

### Construction of gRNA expression vectors and preparation of gRNAs and Cas9 mRNA

Target sequences of gRNAs were selected by using a web program CRISPRdirect (https://crispr.dbcls.jp/) [43]. DNA oligonucleotides, which have targeting sequences with *Bsa*I cutting sites at 5′end were synthesized by Fasmac Co., Ltd. (Atsugi, Japan) and were annealed. The annealed oligonucleotides were cloned into *Bsa*I site of a gRNA expression vector pDR274 [44]. The oligonucleotide sequences for gRNA constructs were shown in S9 Table. gRNAs were transcribed from the *Dra*I-digested gRNA expression vectors as templates by using MEGAshortscript kit (Invitrogen AM1354). The Cas9 mRNA was transcribed from *Sal*I-digested Cas9 expression vector, pSP64-hCas9, by using mMESSAGE mMACHINE SP6 Transcription Kit (Invitrogen AM1340). Following completion of transcription, the samples were treated by DNase I according to the manufacturer’s instructions. Both the gRNA and the Cas9-encoding mRNA were purified by MEGA clear Transcription Clean-Up Kit (Invitrogen AM1908). Purified RNAs were concentrated by ethanol precipitation and re-dissolved in Opti-MEM I Reduced Serum Medium (Gibco 31985062).

### Generating *XmiR*-deficient mice by genome editing

Fertilized eggs were collected from B6D2F1 females mated with Oct4-deltaPE-GFP transgenic male mice in mWM medium (ARK Resource), and were washed with Opti-MEM I Reduced Serum Medium (Gibco 31985062) three times. Eggs were then subjected to electroporation according to a described method [45]. Eggs were aligned in a line in an electrode chamber filled with Opti-MEM I Reduced Serum Medium containing 400 ng of Cas9 mRNA and 200 ng of gRNA (total 5 μl). A condition of electroporation was at 30 V (3 msec ON + 97 msec OFF) × 7 times. After electroporation, the eggs were immediately collected from the electrode and washed with mWM medium three times, and then cultured overnight in mWM medium at 37°C and 5% CO2 incubator. Two-cell embryo were collected and transplanted to pseudopregnant MCH mice.

### Prediction of target genes of XmiRs

Candidate target mRNAs of each XmiR and of neighboring miRNA were predicted by the three web programs (miRDB (http://www.mirdb.org), TargetScan (http://www.targetscan.org), and microT-CDS (http://diana.imis.athena-innovation.gr/DianaTools/index.php?r=microT_CDS/)) based on complementarity of sequences between miRNAs and 3’-UTR of mRNAs[46–48]. Candidate target mRNAs selected by at least one program were applied for further analysis of hierarchical clustering based on similarity of their target sequences by hclust in R program. To predict common target mRNAs of miR-871-3p and miR-880-3p, their target mRNAs predicted by at least the two programs were selected, and common mRNAs between target mRNAs of miR-871-3p and miR-880-3p were identified.

### Histological examination

Testes were fixed in Bouin at 4°C overnight. The fixed testes were dehydrated in a graded series of ethanol (70% to 100%), cleared in xylene, and embedded in paraffin wax. Embedded tissue samples were sectioned at a thickness of 5 μm and then mounted on slides. These sections were deparaffinized with xylene two times, rehydrated with a graded series of ethanol (100% to 70%) and distilled water, and then stained with hematoxylin for 10 min. Sections were rinsed with water for 20 min, stained with 1% eosin for 5min, and then rinsed with water briefly. These sections were dehydrated with a graded series of ethanol (70% to100%) followed by xylene two times and mounted with Permount (Falma). Diameter of seminiferous tubules that were round or nearly round were measured by using Image-J software.

### Immunostaining

Testes of WT and *ΔXmiRs* mice were fixed with 2% paraformaldehyde for overnight and embedded with Optimum Cutting Temperature (O.C.T.) compound (Sakura Finetek 4583). The embedded samples were sectioned using Cryostat CM1900 (Leica) with a section thickness of 10 μm. The sectioned samples were permeabilized and blocked in 5% bovine serum albumin (BSA) and 1% Triton X-100 in PBS for 1 h at room temperature. The sections were then incubated with the primary antibodies diluted by 1% BSA and 0.1% Triton X-100 in PBS overnight at 4°C, and were incubated with the secondary antibodies in the same buffer with 1 μg/ml DAPI for 2 h at 4°C. Samples were washed for 5 min × 3 by 0.1% Triton X-100 in PBS after the primary and the secondary antibody treatments. Samples were mounted with VECTASHIELD (VECTOR H-1000) and observed with confocal laser scan microscope TCS SP8 (Leica). The primary antibodies were: SCP3 (abcam ab15093, 1:100), PLZF (Santa Cruz Biotechnology sc-28319, 1:50), γH2AX (Upstate 05–636, 1:100), Fzd4 (abcam ab83042, 1:100) and β-catenin (Cell signaling technology #8480, 1:100). The secondary antibodies were Goat anti-mouse secondary antibody, Alexa Fluor 647 (Invitrogen A-21235 1:500), Goat anti-mouse secondary antibody, Alexa Fluor 568 (Invitrogen A-11031 1:500), Goat anti-rabbit secondary antibody, Alexa Fluor 568 (Invitrogen A-11011 1:500) and Goat anti-rabbit secondary antibody, Alexa Fluor 488 (Invitrogen A-11034 1:500). Images were acquired under a Leica TCS SP8 confocal microscope. For quantification of β-catenin expression, the Leica Application Suite X program (Leica microsystems, Buffalo Grove, IL, USA) was used for analyzing pixel intensity for β-catenin in a region of interest (ROI) after background subtraction. Mean of pixel intensities in nucleus or cytoplasm of a germ cell was normalized by that of one to three Leydig cells of the same fields. The definition of sub-stages of spermatocytes in prophase I was based on localized patterns of SCP3 [49].

### Total RNA isolation, semi-quantitative RT-PCR of miRNAs and Quantitative RT-PCR

Total RNA containing small RNAs was purifies by using the miRNeasy kit (Qiagen 217004), according to the manufacturer's instructions. For semi-quantitative RT-PCR of miRNAs, total RNA was polyadenylated for 15 min at 37°C in a 5 μl reaction mixture containing 2 U poly(A) polymerase (New England BioLabs M0276). 5 μl volume of polyadenylated RNA was then incubated at 65°C for 5 minutes with 3 pmol oligo(dT)-RACE primer and 1μl 10 mM dNTPs (Roche 11969064001) in a reaction volume of 13 μl. Following addition of 200 U SuperScript III (Invitrogen 18080051), 1 μl RNasin Plus RNase Inhibitor (Promega N2611), 1 μl 0.1 M DTT and 4 μl 5X RT buffer, the reaction was incubated at 50°C for 1 hour followed by 70°C for 15 minutes. Semi-quantitative RT-PCR reaction was performed with Power SYB Green PCR Master Mix (Applied Biosystems 4367659) in a 20 μl reaction containing 5 pmol forward and reverse primer, 0.1 μl cDNA template and 10 μl Power SYBR Green PCR Master Mix and the cycling conditions; 50°C for 2 min, 95°C for 10 min; 40 cycles (95°C, 15 sec; 60°C, 1 min). The amplified products were separated on a 3% agarose gel and visualized by ethidium bromide staining. U6 snRNA was used as an internal control. For quantitative RT-PCR, 5 μl volume of total RNA was incubated at 65°C for 5 minutes with 0.3 μl of Random primers (Promega C118A) and 1μl 10 mM dNTPs (Roche 11969064001) in a reaction volume of 13 μl. Following addition of 200 U SuperScript III (Invitrogen 18080051), 1 μl RNasin Plus RNase Inhibitor (Promega N2611), 1 μl 0.1 M DTT and 4 μl 5X RT buffer, the reaction was incubated at 50°C for 1 hour followed by 70°C for 15 minutes. PCR reaction were performed with Power SYB Green PCR Master Mix (Applied Biosystems 4367659) in a 20 μl reaction containing 5 pmol forward and reverse primer, 0.1 μl cDNA template and 10 μl of Power SYBR Green PCR Master Mix. PCR signals were detected using CFX Connect (Bio-Rad) and the cycling conditions; 50°C for 2 min, 95°C for 10 min; 40 cycles (95°C, 15 sec; 60°C, 1 min). *Arbp* was used as an internal control. All primers used in this study were listed in S9 Table.

### Luciferase reporter assay

Full length 3’UTR of *Fzd4* gene was amplified from mouse genomic DNA by PCR, cloned into an *Xho*I-*Not*I site downstream of a luciferase reporter gene in psiCHECK2 vector (Promega C8021). 1–927 or 927–2263 region of *Fzd4* 3’UTR were amplified from a cloned WT *Fzd4* 3’UTR. For *Fzd4* 3’UTR delta, a set of over-lapping oligo-DNA primers were designed around a target site of the miRNA, in which the target sequence itself is deleted, in *Fzd4* 3’UTR. Fragments of Fzd4 3’UTR up-stream and down-stream to the target site were amplified from a cloned WT *Fzd4* 3’UTR by using the target site primers and outer primers. PCR products were then annealed in the overlapping primer regions and amplified full length *Fzd4* 3’UTR in which the target sites were deleted, by using the outer primers. *miR-871* and *miR-880* genes were amplified from mouse genomic DNA by PCR, cloned into CSII-EF-MCS vector [50] by using In-Fusion cloning kit (Clontech 639648). A luciferase reporter vector and a miRNA expression vector were co-transfected in HEK293T human embryonic kidney cells. HEK293T cells were maintained in DMEM (Gibco 11965092) supplemented with 10% FBS. Luciferase activity was measured by using Dual-Luciferase Reporter Assay System (Promega E1910) 48 hour after transfection. The synthetic Renilla luciferase activity was normalized to synthetic firefly luciferase activity for each sample.

### Purification of stage-specific spermatogenic cells by FACS sorting

Dissociation of testicular cells and subsequent staining was carried out based on a described method [51]. Testes were dissected from B6 mice at 10 to 12 weeks of age. After removing albuginea, testes were incubated for 25 min in 6 ml of Gey’s Balanced Salt Solution (GBSS; Sigma-Aldrich G9779) containing 1.2 mg/ml of Collagenase Type I (Sigma-Aldrich C0130) at 32°C, and seminiferous tubules were dissociated. Interstitial cells were removed by filtration with a 40 μm Cell strainer (Falcon 352340). Seminiferous tubes retained on the filter were collected and incubated at 32°C for 25 min in the same collagenase-containing buffer as that used for the first step. Cell aggregates were sheared gently by 10 times of pipetting with wide orifice plastic transfer pipet and filtered through 40 μm Cell strainer to remove cell clumps. Cells were washed with GBSS and then resuspended in GBSS containing 1% FBS. Two million cells were diluted in 2 ml of GBSS containing 1% FBS and stained with 5 μg/ml of Hoechst 33342 (Invitrogen H3570) for 1 h at 32°C. Cells were kept on ice and protected from light until sorting. Before sorting, 10 μl of propidium iodide (Becton-Dickinson 51-66211E) was added to the stained cells and filtered through 40 μm Cell strainer. Sorting was performed on a Becton-Dickinson FACSAria II cell sorter. Total RNA was purified from 1 x10^6^ cells of round spermatids and 1 x 10^5^ cells of other population in testes by using the miRNeasy kit (Qiagen 217004), according to the manufacturer's instructions.

### Overexpression of miR-871, miR-880 and stable β-catenin in GSCs

miR-871 and miR-880 genes were amplified from mouse genomic DNA by PCR, cloned into an *Age*I-*Eco*RI site of pLKO1 vector [52]. For construction of stable β–catenin, amino acid substitutions of S33A, S37A, T41A, and S45A to prevent phosphorylation by GSK3 [53] were introduced. A set of over-lapping oligo-DNA primers were designed around the mutation site. Fragments of stable β–catenin up-stream and down-stream to the mutation site were amplified from mouse cDNA by using the target site primers (up-stream: Ctnnb1-AAAA-Rev, down-stream: Ctnnb1-AAAA-Fw) and outer primers (up-stream: Kozak-Ctnnb1-Fw-for-CS2-EF-MCS, down-stream: Ctnnb1-Rev-for-CS2-EF-MCS). An up-stream fragment of stable β–catenin was annealed to Ctnnb1 AAAA AS oligo, which is partially overlapped with Ctnnb1-AAAA-Rev and Ctnnb1-AAAA-Fw. It was then amplified with Kozak-Ctnnb1-Fw-for-CS2-EF-MCS primer, which results in an elongated up-stream fragment covering all mutation sites. The elongated up-stream fragment was then annealed with the downstream fragment in the overlapping regions, and full length stable β–catenin was amplified by using the outer primers (Kozak-Ctnnb1-Fw-for-CS2-EF-MCS and Ctnnb1-Rev-for-CS2-EF-MCS). PCR product was cloned into CSII-EF-MCS vector [50] by using In-Fusion cloning kit (Clontech 639648). Lentivirus particles were produced as described previously. Briefly, pLKO1 (miR-971, miR-880)- or CSII-EF-MCS (stable-β–catenin)-lentivirus vector, pCMV-VSV-G-RSV-Rev and pCAG-HIVgp [50] were co-transfected into HEK293T cells by the calcium phosphate method. After 48 hours, cell supernatant containing lentiviral particles was collected, and virus concentration was determined by using Lenti-X qRT-PCR Titration Kit (Clontech 631235) according to the manufacturer’s instructions. Virus particles were collected by centrifuging the cultured medium at 2,330 × g for 30 minutes at 4°C after incubating with PEG6000 solution [final 2.5% PEG6000 (Wako), 100 mM NaCl, 10 mM HEPES (pH 7.4)] overnight at 4°C, and they were re-suspended in GS medium [54] (StemPro-34 SFM (Gibco 10640–019) supplemented with StemPro-34 Nutrient Supplement (Gibco 10641–025), 25 μg/ml insulin, 100 μg/ml transferrin, 60 μM putrescine (Sigma P5780), 30 nM sodium selenite (Sigma S9133), 6 mg/ml D-(+)-glucose (Gibco A24940), 30 μg/ml pyruvic acid (Gibco 11360070)), 1 μl/ml DL-lactic acid (Sigma 69785), 5 mg/ml bovine albumin (Sigma A3311), 2 mM L-glutamine, 50 μM 2-mercaptoethanol (Sigma M3148)), 1 x minimal essential medium (MEM) vitamin solution (Gibco 11120052), 1 x MEM nonessential amino acid solution (Gibco 11140050), 100μM ascorbic acid (Sigma A4544), 10 μg/ml d-biotin (Sigma B4639), 30 ng/ml β-estradiol, 60 ng/ml progesterone (Sigma P6149), 20 ng/ml mouse epidermal growth factor, 10 ng/ml human basic fibroblast growth factor (Sigma F0291), 10 ng/ml recombinant rat glial cell line-derived neurotrophic factor (GDNF) and 1% fetal calf serum). GSCs (DBAGS) [54] were cultured in GS medium on MEF feeder cells. Infection of the lentivirus vectors to GSCs were carried out as described previously with some modifications. Lentivirus was infected to GS cells at 1 x 10^4^ lentiviral particle/cell [55] and incubated at 37°C with 5% CO_2_ overnight. The culture medium containing the transfection mixture was discarded and replaced with GS culture medium containing 500 ng/ml puromycin 16–24 h after infection. GSCs were collected by trypsin treatment at day 4, 6, 8 and 10 after the virus infection and cell number were counted. GSCs were collected by trypsin treatment at day 8 and total RNA was purified by using the miRNeasy kit (Qiagen 217004), according to the manufacturer's instructions. All primers used in this study were listed in S9 Table.

## Results

### Identification of germ cell-specific miRNAs

**Fig 1:**
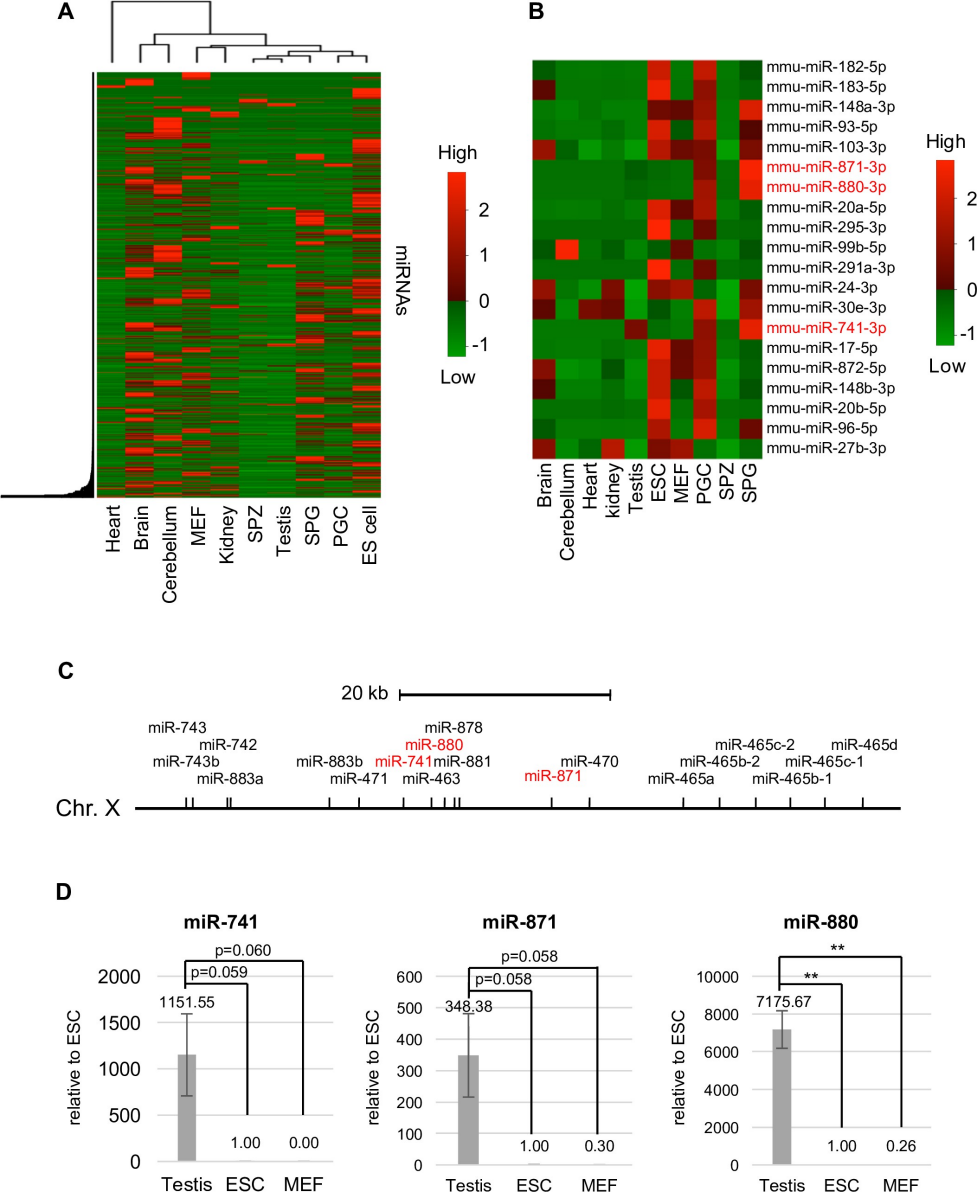
The expression profile of miRNAs in various tissues and cell lines. (A) A heat map of hierarchical clustering of miRNAs detected in small RNA-seq data used in this study. (B) A heat map of 20 miRNAs highly expressed in PGCs. Relative miRNA expression is described according to the color scale. Red and green indicate high and low expression, respectively. Mouse embryonic fibroblasts (MEFs), embryonic stem (ES) cells, primordial germ cells (PGCs), spermatogonia (SPG), spermatozoa (SPZ). (C) The locus of *XmiR* genes on the X chromosome. (D) The expression of XmiRs in testes, ES cells, and MEFs determined by quantitative RT-PCR. Each expression level was normalized to the expression of U6 snRNA. The expression in ES cells was set as 1.0. Error bars show standard errors of three biological replicates. **P < 0.01.

To identify germ cell-specific miRNAs, we compared the expression of miRNAs among somatic tissues, cell lines, and germ cells by using published small RNA-seq data of mouse [40–42]. The proportion of miRNAs to the total reads in germ cells was less than that in somatic tissues (S1 Table). In addition to miRNAs, other small RNAs, such as Piwi-interacting (pi) RNAs that maintain genomic quality were contained in the small RNA-seq data of germ cells [56]. Cluster analysis showed that the expression profiles of these miRNAs were classified into three groups, i.e., those highly expressed in somatic tissues or mouse embryonic fibroblasts (MEFs), in spermatogenic cells, and in PGCs and embryonic stem (ES) cells (Fig 1A). Of the 20 most abundantly expressed miRNAs in PGCs, many miRNAs were also highly expressed in ES cells (Fig 1B and S2 Table). miR-741-3p, miR-871-3p, and miR-880-3p were highly expressed in PGCs and spermatogonia, but were rarely expressed in ES cells or somatic tissues. The results suggest that these miRNAs have germ cell-specific functions. Interestingly, these miRNA genes were located on the X chromosome in contiguity with other miRNA genes (Fig 1C). Their high expression in testes was confirmed with RT-qPCR (Fig 1D). We named these three miRNAs X chromosome-linked miRNAs (XmiRs).

### Defective spermatogenesis in *XmiR*-deficient mice

**Fig 2:**
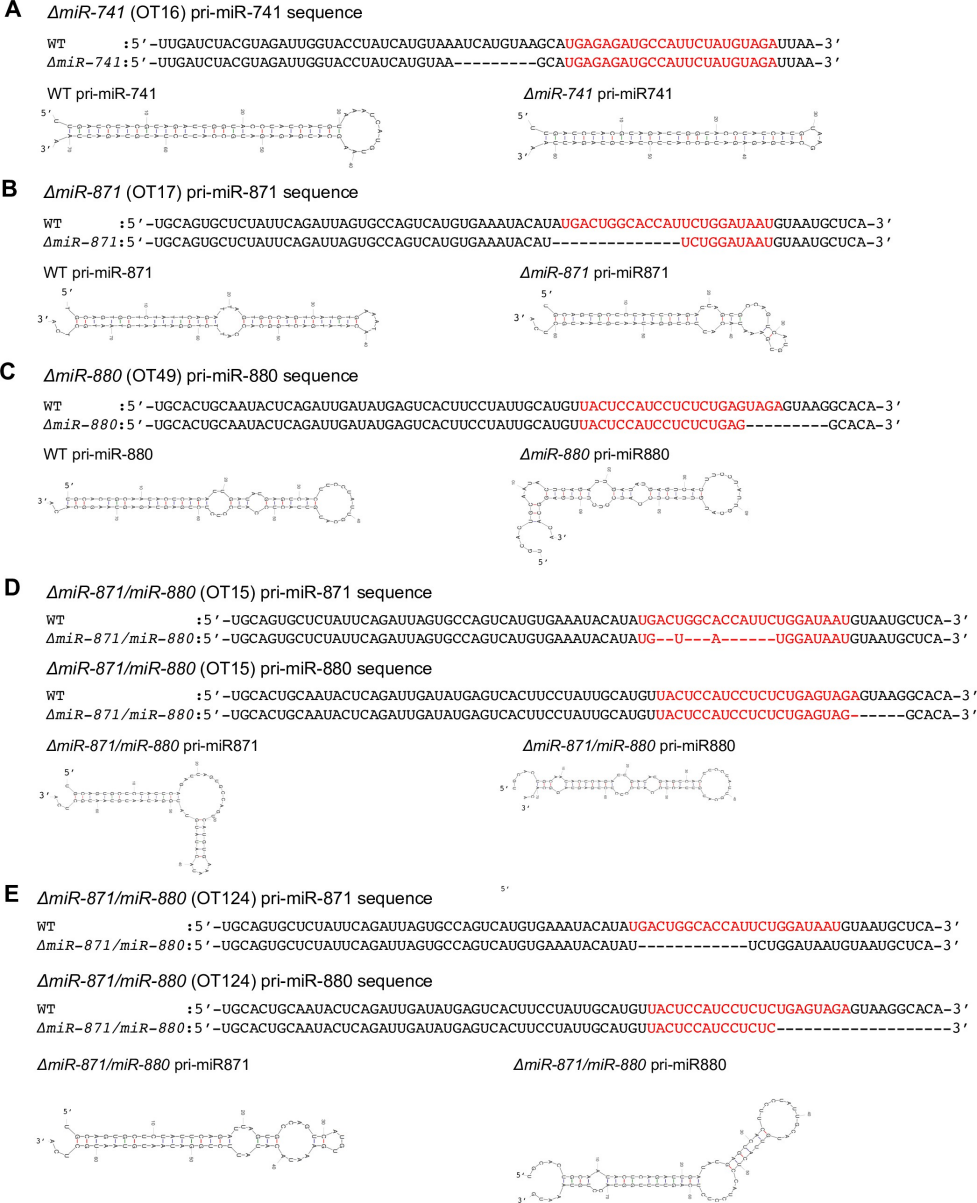
Predicted altered secondary structures of XmiR precursors by genome editing. Secondary structures of predicted miRNA precursors of (A) miR-741 WT (left) and *ΔmiR-741* (OT16) (right), (B) miR-871 WT (left) and *ΔmiR-871* (OT17) (right), (C) miR-880 WT (left) and *ΔmiR-880* (OT49) (right), and (D, E) miR-871 (top) and miR-880 (bottom) in *ΔmiR-871/ΔmiR-880* in two different mice, OT15 (D) and OT124 (E).

**Fig 3:**
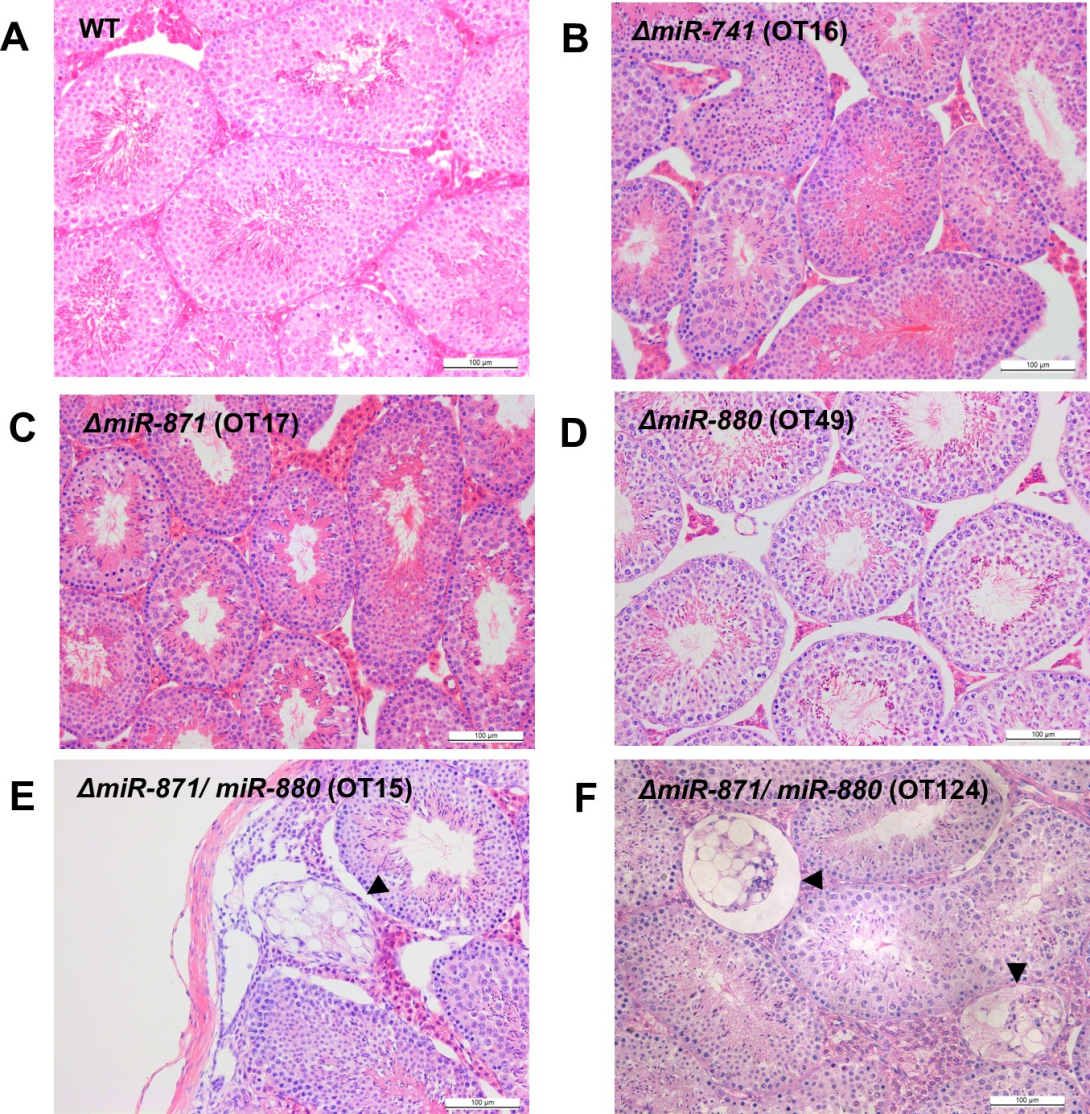
Spermatogenesis in mice with mutations in *miR-741*, *miR-871*, and *miR-880*. Hematoxylin-eosin (HE)-stained sections of seminiferous tubules in the testes of (A) WT, (B) *ΔmiR-741* (OT16), (C) *ΔmiR-871* (OT17), (D) *ΔmiR-880* (OT49), and *ΔmiR-871/ΔmiR-880* (E) (OT15) mice at 8 weeks of age, and (F) *ΔmiR-871/ΔmiR-880* (OT124) mouse at 24 weeks of age. The arrowheads show abnormal seminiferous tubules. Scale bars = 100 μm.

To examine the functions of *XmiRs* in germ cells, we generated knockout mice for each *XmiR* gene with the CRISPR/Cas9-mediated genome editing system [57]. gRNAs were designed in the Dicer processing site [58], seed sequence [59], and Drosha processing site [60] for *miR-741*, *miR-871*, and *miR-880*, respectively. In the *ΔmiR-741* mouse, a deletion occurred in the Dicer processing site that shortened the predicted terminal loop and stem. Drosha has a strong preference for pri-miRNA hairpin structures with a large (>10 nucleotides) terminal loop, suggesting that this deletion causes a severe defect in miR-741-3p production (Fig 2A) [61]. In the *ΔmiR-871* mouse, the seed sequence was completely deleted, suggesting that miR-871-3p cannot recognize its target mRNA (Fig 2B) [59]. In the *ΔmiR-880* mouse, the Drosha processing site was deleted, and the lower stem that binds to DGCR8 was shortened, suggesting that the *ΔmiR-880* mouse cannot generate functional pre-miRNA (Fig 2C) [62]. Testis weight (S1A Fig) and spermatogenesis evaluated by histological analysis (Fig 3A–3D) revealed no differences between wildtype (WT) and mutant testes in which a single *XmiR* gene was deficient, though seminiferous tubules were thinner in testes of *ΔmiR-741* and *ΔmiR-871* (S1B Fig). Among these *XmiR* gene-deficient mice, we obtained two mice (OT15 and OT124) in which *miR-871* and *miR-880* were doubly mutated by chance due to loss of the seed sequence in miR-871-3p and shortening of the hairpin structure of miR-880-3p due to deletion of the Drosha target site (Fig 2D and 2E). These two mice showed abnormal spermatogenesis in a few seminiferous tubules at 8 and 24 weeks of age (Fig 3E and 3F), suggesting that *XmiRs* act in a functionally redundant manner.

**Fig 4:**
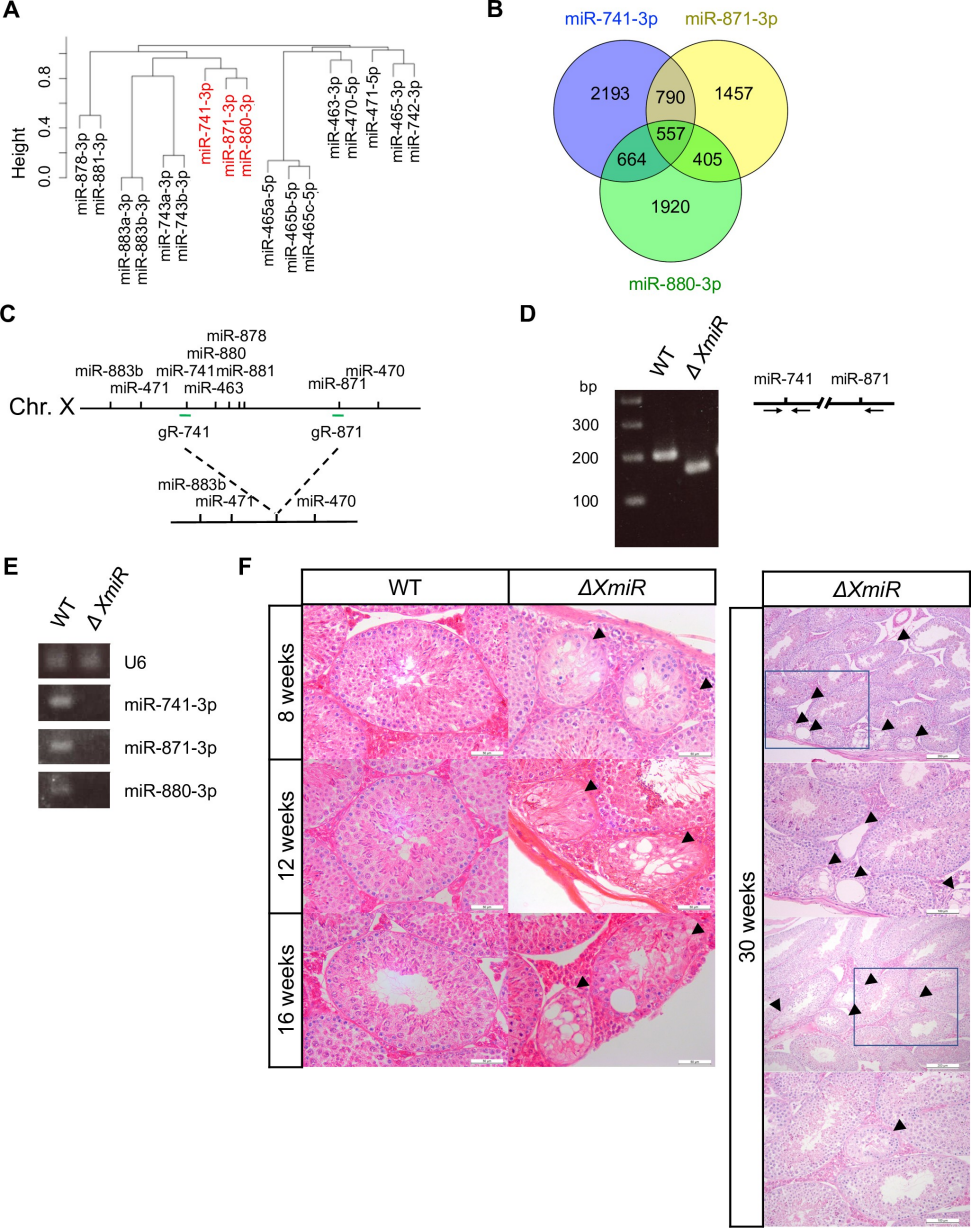
Relationship between target mRNAs of XmiRs and generation of *ΔXmiRs* mice. (A) A dendrogram of hierarchical clustering analysis of target mRNAs of XmiRs and their neighboring miRNAs. (B) Venn diagram showing the relationship among putative target mRNAs of miR-741-3p, miR-871-3p, and miR-880-3p. Corresponding gene lists are shown in S3 Table. (C) A schematic presentation of the WT and *ΔXmiRs* locus. gR-741 and gR-871 represent positions of guide RNAs used for genome editing. (D) Representative PCR for genotyping of WT and *ΔXmiRs* (OT84) mice. Arrows in the right panel represent primers used for PCR. (E) Expression of XmiRs in WT and a *ΔXmiRs* testes (F2 of OT84) determined with semi-quantitative RT-PCR analysis. U6 snRNA was used as an internal control. (F) HE-stained sections of seminiferous tubules in WT (left) and *ΔXmiR* (F2 of OT100) (right) testes at 8, 12, 16, and 30 weeks of age. The second and fourth panels for 30 weeks show higher magnification views corresponding to the rectangular area in the first and third panels. Lower two panels show mildly affected seminiferous tubules. Arrowheads show abnormal seminiferous tubules. Scale bar = 50 μm (8, 12, 16 weeks), 200 μm (30 weeks, lower magnification), 100 μm (30 weeks, higher magnification).

To test this possibility, we examined target mRNAs of each XmiR and other miRNAs nearby shown in Fig 1C by using three web programs (miRDB (http://www.mirdb.org), TargetScan (http://www.targetscan.org), and microT-CDS (http://diana.imis.athena-innovation.gr/DianaTools/index.php?r=microT_CDS/)) that predict targets of miRNAs by evaluating each potential miRNA-target mRNA pair based on complementarity of a seed sequence and target sequences [46–48]. Hierarchical clustering analysis showed that the predicted target mRNAs of XmiRs converged on the same clusters (Fig 4A), and a portion of their target mRNAs actually overlapped (Fig 4B and S3 Table).

To further investigate the functional redundancy of XmiRs, we generated mutant mice with a large deletion covering all three *XmiR* genes (*ΔXmiRs*) (Fig 4C and 4D). We confirmed that *ΔXmiRs* region did not contain any protein coding genes. We obtained three independent mutant mouse lines (OT84, OT87, and OT100), which showed identical testicular abnormalities. Expression of the XmiRs was not detected in the testes of a *ΔXmiRs* mouse with RT-PCR (Fig 4E). *ΔXmiRs* mice grew normally, and their fertility was indistinguishable from that of WT or heterozygous littermates (S4 Table). As in mice with deficiency of a single *XmiR*, testis weight was not affected but seminiferous tubules were thinner in *ΔXmiRs* compared with those in WT (S1C and S1D Fig). However, in the testes of *ΔXmiRs* mice, a few abnormal seminiferous tubules were observed at 8 weeks of age, and the number of defective seminiferous tubules increased with age (Fig 4F and S5 Table). Similar to *miR-871/miR-880* doubly mutated mice, fewer germ cells were present in the abnormal seminiferous tubules. In the severely affected seminiferous tubules, germ cells were rarely observed, while a few germ cells were found in the mildly affected tubules (Fig 4F, 30 weeks).

**Fig 5:**
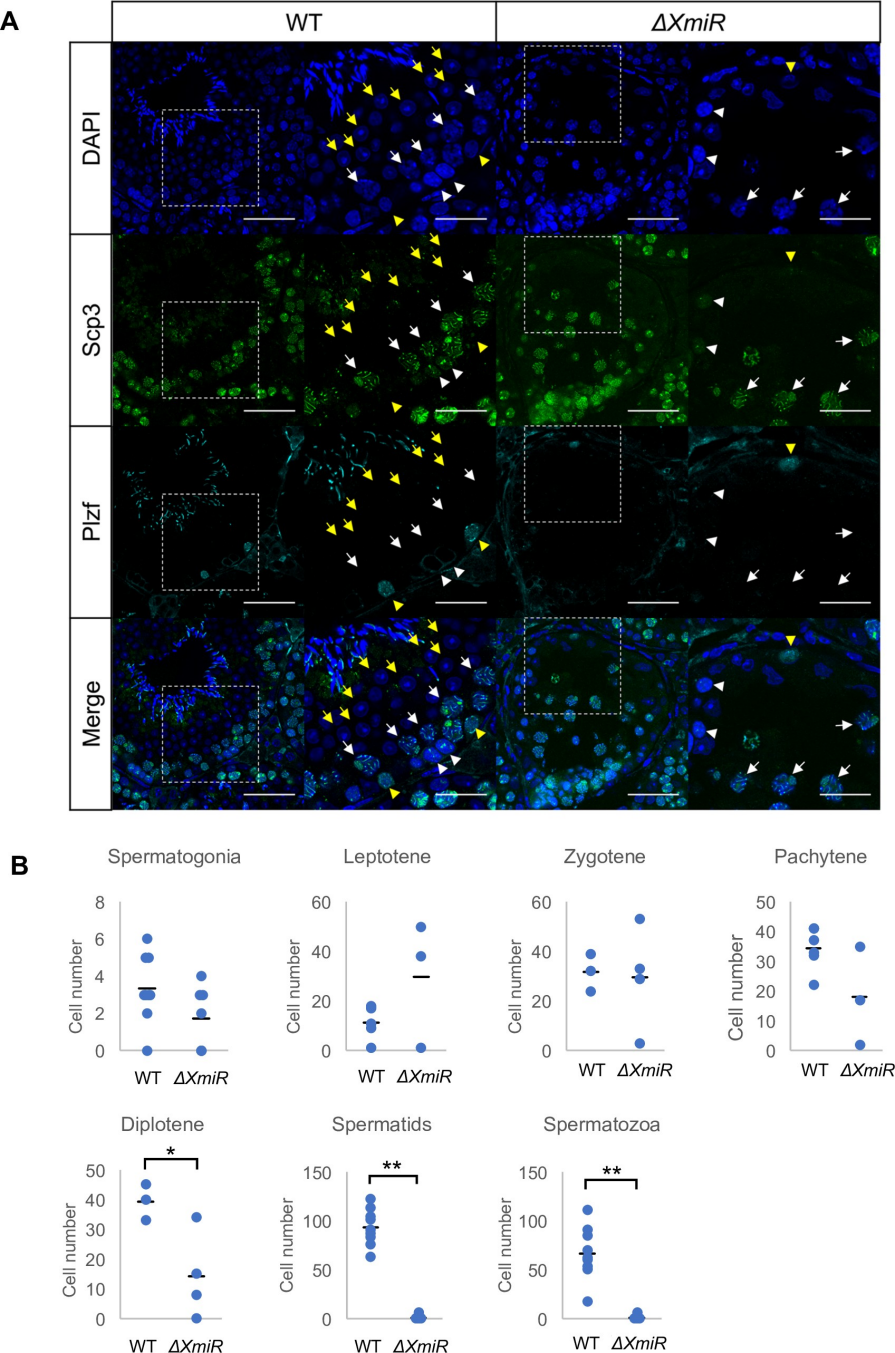
Spermatogenesis was arrested at meiotic prophase in abnormal seminiferous tubules in *ΔXmiR* testes. (A) Testis sections were co-stained with anti-SCP3 (green) and anti-PLZF (cyan) antibodies in WT and *ΔXmiRs* (F2 generation of the OT100 line) mice at 12 weeks of age. The second and fourth column show higher magnification views corresponding to the rectangular area in the pictures in the first and third columns. Mildly affected seminiferous tubules in *ΔXmiR* testis shown in Fig 4F are presented. Spermatogonia (yellow arrowheads), leptotene spermatocytes (white arrowheads), pachytene spermatocytes (white arrows), and round spermatids (yellow arrows) are indicated. Scale bars = 50 μm (the first and third columns) and 25 μm (the second, and fourth columns). (B) Number of cells at different spermatogenic stages determined by staining for Scp3 and Plzf in WT and mildly affected seminiferous tubules in *ΔXmiR* testis. Cells in two sections in a single mouse of WT and *ΔXmiR* testes were counted. Vertical lines in the graphs indicate means. *P < 0.05 and **P < 0.01.

To determine the spermatogenic stages affected in *ΔXmiRs* mice, we performed immunostaining of testes of WT and *ΔXmiRs* mice using an antibody against Synaptonemal complex protein 3 (SCP3), a component of the synaptonemal complex [63], and an antibody against Promyelocytic leukemia zinc finger protein (PLZF), which is required for self-renewal of undifferentiated SSCs [64, 65]. Number of SSCs, leptotene, zygotene and pachytene spermatocytes was not significantly changes in the mildly affected seminiferous tubules of *ΔXmiR* mice, but diplotene spermatocytes were decreased, and spermatids which are observed as cells with condensed nuclei following DAPI staining as well as spermatozoa were rarely found (Fig 5A and 5B). We also examined the expression of γH2AX (phosphorylated histone H2AX), and found dot-like signals likely representing localization on XY body in pachytene spermatocytes in *ΔXmiRs* as well as WT testes (S2 Fig). This result suggests that spermatogenesis was arrested in meiosis in the abnormal seminiferous tubules of *ΔXmiR* mice.

### Identification of target genes of XmiRs

Because *ΔXmiRs* mice showed similar abnormalities as those in *miR-871/miR-880* double mutant mice (Figs 3E and 3F and 4F), *miR-871/miR-880* but not *miR-741* are likely important for spermatogenesis. Therefore, we attempted to identify common target genes of miR-871-3p and miR-880-3p. We first selected putative target mRNAs of miR-871-3p and miR-880-3p by using the same web programs used for the analysis in Fig 4A, and chose mRNAs commonly selected by at least two of the three web programs as promising candidates. We consequently identified 46 common target mRNAs of miR-871-3p and miR-880-3p by comparing their targets (S3 Fig and S6 Table).

**Fig 6:**
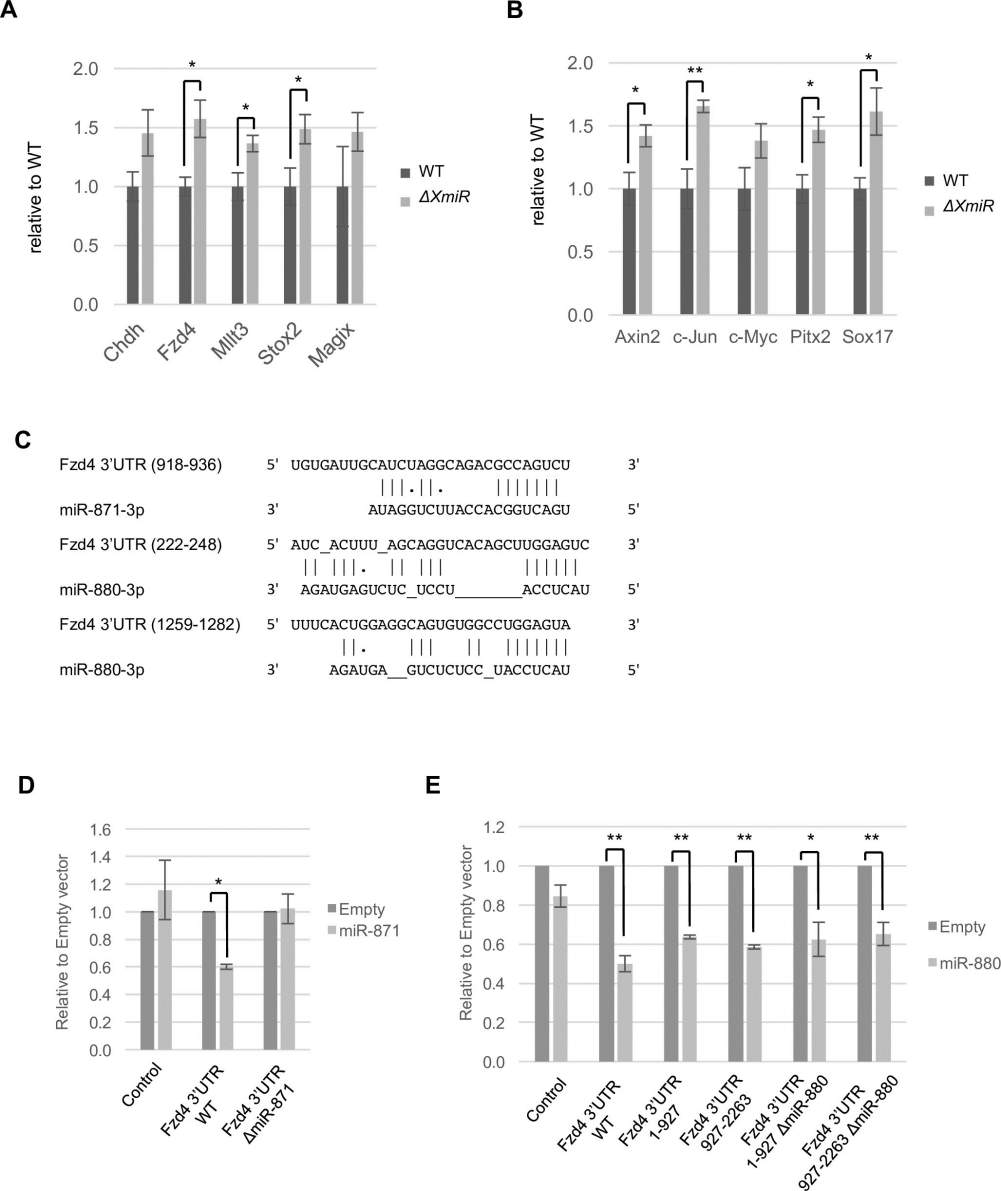
Upregulation of WNT/β-catenin signaling genes in testes of *ΔXmiRs* mice, and repression of *Fzd4* by XmiRs. (A, B) Relative expression of the putative common target genes of miR-871-3p and miR-880-3p (A) and of the downstream genes of WNT/β-catenin signaling (B) in the testes of WT and *ΔXmiRs* (F2 of the OT84 line) mice at 12 weeks of age was determined by quantitative RT-PCR analysis. The expression in WT testis was set as 1. (C) Potential target sites of miR-871-3p and miR-880-3p in Fzd4-3′-UTR. (D, E) Relative luciferase activity from the reporter vectors with Fzd4-3′-UTR with or without expression vectors for miR-871 (D) or miR-880 (E). Fzd4-3′-UTR ΔmiR-871; Fzd4-3′-UTR with deleted miR-871-3p target site (D). Fzd4-3′-UTR 1–927 or 927–2263 with or without ΔmiR-880; 1–927 or 927–2263 bp fragment of Fzd4-3′-UTR with or without deleted miR-880-3p target sites (E). Luciferase activity was measured 48 h after transfection. Luciferase activity with an empty expression vector was set as 1. Error bars represent standard errors of three biological replicates. *P < 0.05 and **P < 0.01.

We next used RT-qPCR to examine the expression of those target genes in the testes of WT and *ΔXmiR* mice. Among the candidates, the expression of *Fzd4*, *Mllt3*, and *Stox2* was significantly increased in the testes of *ΔXmiR* mice compared with that in WT testes, and *Chdh* and *Magix* also tended to be upregulated (Figs 6A and S4). Several studies have reported the contribution of the WNT/β-catenin signaling pathway to spermatogenesis [33–38]. *Fzd4* encodes a WNT receptor. Because no previous reports have shown roles for *Mllt3*, *Stox2*, or *Magix* in spermatogenesis, and the only known function of *Chdh* is involvement in sperm motility [66], we focused on *Fzd4* for further analysis.

Because the expression of *Fzd4* was increased in *ΔXmiRs* mouse testes, the expression of downstream genes of WNT/β-catenin signaling is also likely upregulated in *ΔXmiRs* mouse testes. To investigate this possibility, we used RT-qPCR to examine the expression of previously reported possible downstream genes of WNT/β-catenin signaling in the testes of WT and *ΔXmiR* mice. Four of the five tested downstream genes were significantly increased, and *c-Myc* also tended to be upregulated in *ΔXmiRs* testes (Fig 6B). We also found that the expression of β-catenin protein was increased both in cytoplasm and nucleus in some SSCs and spermatocytes in *ΔXmiRs* testes compared with that in WT testes (S5 Fig). It suggests that β-catenin protein is stabilized to transmit signal in *ΔXmiR* testis. These results suggest that XmiRs repress WNT/β-catenin signaling via repression of *Fzd4* expression.

### Inhibition of luciferase (luc)-Fzd4-3′-UTR reporter gene expression by XmiRs

We performed a luc assay to investigate whether *miR-871* and *miR-880* directly repressed *Fzd4* expression. To predict the miRNA target site, we used DIANA tools in the microT-CDS program (http://diana.imis.athena-innovation.gr/DianaTools/index.php?r=microT_CDS/) [48], and found one and two candidate target sites for miR-871-3p and miR-880-3p, respectively (Fig 6C). Expression vectors for *miR-871* or *miR-880* were co-transfected with a luc reporter containing the Fzd4-3′-UTR into HEK293T cells. *miR-871* and *miR-880* repressed luc activity (Fig 6D and 6E). We then used a luc reporter containing the Fzd4-3′-UTR in which a miR-871-3p target site was deleted (Fzd4-3′-UTR ΔmiR-871). In this case, luc activity was not repressed by *miR-871* (Fig 6D), indicating that miR-871-3p inhibited the expression of the reporter via its target site. Because the Fzd4-3′-UTR has two candidate target sites for miR-880-3p, we co-transfected *miR-880* with luc reporters containing a part of the Fzd4-3′-UTR that has each candidate target site. *miR-880* significantly repressed luc reporters with each fragment of the Fzd4-3′-UTR. However, *miR-880* also repressed luc activity from reporter vectors with Fzd4-3′-UTR fragments in which putative miR-880-3p target sites were deleted (Fzd4-3′-UTR 1–927 ΔmiR-880; Fzd4-3′-UTR 927–2263 ΔmiR-880) (Fig 6E). The results suggest that *miR-880* targets other sequences in the Fzd4-3′-UTR that were not predicted by the program.

### The expression of *XmiRs* and *Fzd4* in spermatogenic cells

**Fig 7:**
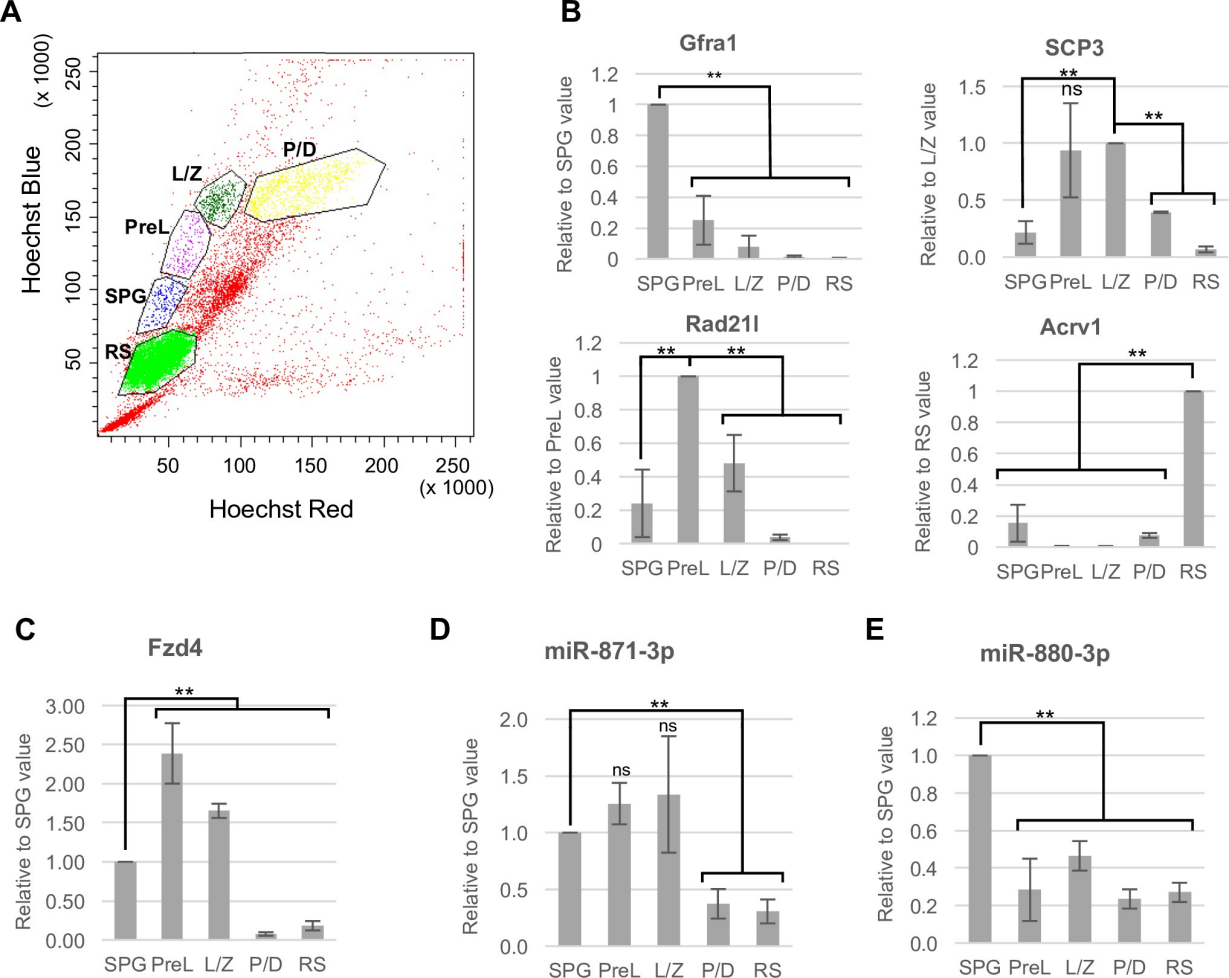
Expression of *XmiRs* and *Fzd4* in spermatogenic cells. (A) Scatter plot of FACS for testicular cells stained with Hoechst 33342. Spermatogonia (SPG), pre-leptotene (PreL), leptotene-zygotene (L/Z), pachytene-diplotene (P/D), round spermatids (RS). (B-E) Relative expression of stage-specific germ cell marker genes (B), *Fzd4* (C), miR-871-3p (D), and miR-880-3p (E) in FACS-purified spermatogenic cells. *Gfra1* for spermatogonia, *Scp3* and *Rad21l* for spermatocytes, *Acrv1* for spermatids. Gene expression was determined by RT-qPCR. Error bars represent standard errors of three biological replicates. **P < 0.01.

To determine the stages of spermatogenic cells in which *miR-871*, *miR-880*, and *Fzd4* are expressed, we purified testicular germ cells at different spermatogenic stages with fluorescence-activated cell sorting (FACS) (Fig 7A). We first confirmed the purity of the sorted germ cells by examining the expression of stage-specific germ cell marker genes (Fig 7B). Spermatogonia-specific *Gfra1* [67], spermatocyte-specific *Scp3* [63] and *Rad21l* [68], and spermatid-specific *Acrv1* [69] showed the expected expression levels in each cell fraction. The expression of Fzd4 mRNA increased from spermatogonia to the preleptotene-zygotene stage and then decreased from the pachytene stage onward (Fig 7C). Meanwhile, FZD4 protein was apparently upregulated in pachytene spermatocytes (S6 Fig), suggesting its translational regulation by XmiRs. On the other hand, miR-871-3p was expressed from spermatogonia to leptotene-zygotene spermatocytes, but its expression decreased after the pachytene stage onward (Fig 7D). miR-880-3p was expressed in spermatogonia, and its expression decreased at the onset of meiosis (Fig 7E). miR-871-3p and miR-880-3p in spermatogenic cells likely play a role in adjusting *Fzd4* expression levels and subsequent WNT/β-catenin signaling levels that are suitable for proper development of spermatogonia and spermatocytes.

### Overexpression of XmiRs represses growth and/or survival of germ stem cells (GSCs)

**Fig 8:**
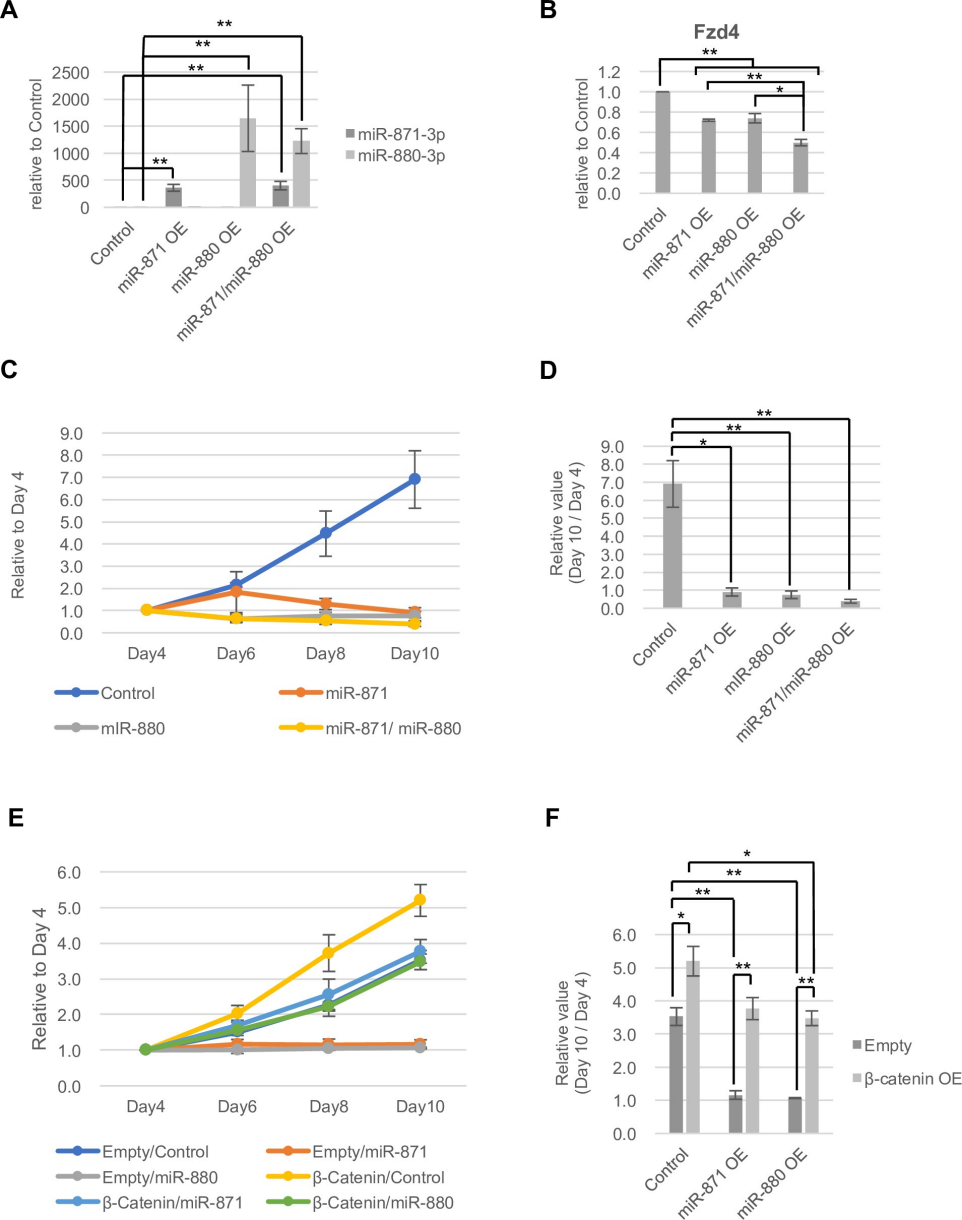
Roles of *miR-871*, *miR-880* and stable β-catenin in GSCs. (A, B) Relative expression of *miR-871* and *miR-880* (A) and *Fzd4* (B) in GSCs overexpressing XmiRs at 8 days after infection with the lenti-virus vectors. Gene expression was determined with RT-qPCR. (C, D) The effect of *XmiRs* overexpression in GSCs in culture. The pLKO1 empty vector was used as the control. Cell number at day 4 to day 10 after infection with the lenti-virus vector (C), and ratios of GSC number at day10 compared with that at day4 (D) are shown. (E, F) The effect of co-overexpression of stable β-catenin and *miR-871* or *miR-880*. The pLKO1 empty vector and CSII-EF-MCS vector were used as the control. Cell number at day4 to day10 after infection with the indicated lenti-virus vectors (E), and ratios of GSC number at day10 compared with that at day4 (F) are shown. Error bars represent standard errors of three biological replicates. *P < 0.05 and **P < 0.01.

To examine functions of XmiRs in SSCs, we overexpressed XmiRs in a GSC line in culture [54]. We confirmed that the expression of *miR-871* and *miR-880* was upregulated at 8 days after infection of lenti-virus vectors (Fig 8A). The expression of *Fzd4* was repressed by overexpression of *miR-880* and/or *miR-871*, and those miRNAs additively repressed *Fzd4* expression (Fig 8B). The number of GSCs was unchanged when expression vectors of miR-871-3p and/or miR-880-3p were infected, whereas GSCs increased in number with an empty vector (Fig 8C and 8D). The results suggest that excess miR-871-3p and miR-880-3p repress proliferation and/or survival of GSCs.

We then tested whether WNT/β-catenin signaling was under the control of XmiRs in GSCs. We co-transfected an expression vector of stable β-catenin [53] with an expression vector of miR-871 or miR-880. Stable β-catenin alone enhanced GSC number, indicating that WNT/ β-catenin signaling stimulates proliferation and/or survival of GSCs (Fig 8E and 8F). In addition, GSCs also increased in number by stable β-catenin with the expression of miR-871 or miR-880. Although decreased GSCs by miR-871 or miR-880 were not completely recovered by stable β-catenin, ratios of increased GSCs by stable β-catenin with miR-871 or miR-880 was higher compared with those with a control XmiR vector (S7 Table). It suggests that stable β-catenin partially rescues the influence of the XmiRs in GSCs, implying that WNT/β-catenin signaling functions under the control of XmiRs.

## Discussion

In this study, we identified miR-741-3p, miR-871-3p, and miR-880-3p as being highly and preferentially expressed in germ cells. The genes for these three miRNAs are clustered on the X chromosome. In mice, 28.1% of miRNA genes form clusters on various chromosomes [70]. miRNA gene clusters may arise by de novo formation of miRNA-like hairpin structures in existing primary miRNA transcript units or by tandem duplication of a single miRNA gene [71]. *XmiRs* and additional miRNA gene clusters nearby may also be generated by similar molecular mechanisms.

A single mRNA is likely targeted by multiple miRNAs due to the short length of seed sequences. Several studies proposed that miRNAs in the same gene cluster are often functionally correlated, and those miRNAs exert cooperative and/or redundant repressive effects on the same target genes. For example, members of the *miR-17-92* gene cluster are highly conserved among vertebrates, and cooperatively regulate transforming growth factor (TGF)-β signaling and cell cycle regulation; *miR-17* and *miR-20a* directly target TGF-β receptor II mRNA, whereas *miR-18a* targets the mRNA of Smad2 and Smad4, two members of the TGF-β signaling pathway [72]. In addition, *miR-17* and *miR-20a* cooperatively modulate the expression of E2F1 [73], a member of the E2F family of transcription factors that play a central role in the regulation of G1 to S phase progression [74]. *miR-20a* also targets both E2F2 and E2F3 [75]. In the case of *XmiRs*, we found that *miR-871* and *miR-880* had redundant functions in spermatogenesis via repression of the expression of a WNT signaling molecule, FZD4. *ΔXmiR* mice showed subtle abnormalities in spermatogenesis. Although the expression was upregulated in *ΔXmiRs* testes, the increase of *Fzd4* expression was marginal (Fig 6A). The results together suggest that additional miRNAs redundantly target *Fzd4*. Consistent with this idea, we found other miRNAs in addition to XmiRs, which are predicted to target the *Fzd4* 3′-UTR, among miRNAs expressed in testes (S7 Fig and S8 Table).

WNT/β-catenin signaling is an important pathway involved in proliferation and differentiation of stem cells in many different tissue types including testicular germ cells. Regarding the WNT receptor gene in testis, *Fzd2*, *Fzd3*, *Fzd7*, and *Fzd8* are expressed in spermatogonia [37], whereas we found that *Fzd4* was expressed in spermatogonia, spermatocytes, and spermatids (Fig 7C), suggesting functions for FZD4 in those cells. Previous *in vitro* studies have shown that WNT3A and WNT5A promote proliferation of SSCs [33, 34]. Consistent with this observation, conditional knockout of the β-catenin gene in testicular germ cells with *Axin2-Cre* suppresses proliferation of undifferentiated spermatogonia [35], and we also consistently found that stable β-catenin enhanced GSC number in culture (Fig 8E and 8F). However, WNT/β-catenin signaling-activated SSCs show reduced SSC activity after transplantation into seminiferous tubules [34]. Constitutive activation of β-catenin expression in testicular germ cells in transgenic mice causes progressive loss of spermatocytes and spermatids and reduction of meiotic germ cells from the leptotene to pachytene stages [38]. Taken together, the results indicate that WNT/β-catenin signaling enhances proliferation of SSCs, but represses their differentiation.

We showed that XmiRs targeted Fzd4 mRNA and downstream genes of WNT/β-catenin signaling were upregulated in *ΔXmiR* testes (Fig 6A and 6B) in which a few spermatocytes but no spermatids were observed in some seminiferous tubules (Fig 5). The results together suggest that deficiency in XmiRs results in abnormal enhancement of WNT/β-catenin signaling in spermatogenic cells, which may cause impaired meiosis. Whether or not X-linked miRNAs escape meiotic sex chromosome inactivation (MSCI) is controversial [76–78], but we found the expression of miR-871-3p was downregulated in pachytene/diplotene spermatocytes (Fig 7D), suggesting that it suffers silencing at least in some extent. Meanwhile, sex bodies associating with MSCI was observed in remaining pachytene spermatocytes in *ΔXmiR* testes (S2 Fig), suggesting MSCI occurs in *ΔXmiR* spermatocytes.

Although the testicular abnormalities in *ΔXmiR* mice were subtle, the mice showed progressively more severe testicular abnormalities with age (Fig 4F and S5 Table). Constitutive activation of β-catenin in testicular germ cells in transgenic mice results in progressive loss of spermatogenic cells, in which fewer than 5% of seminiferous tubules are defective in spermatogenesis at 13 weeks of age. The ratio of defective tubules increases to more than 40% of total tubules at 75 weeks of age [38]. These data suggest that the influences of abnormal activation of WNT/β-catenin signaling by XmiR deficiency may gradually accumulate in some germ cells with aging. Those germ cells may undergo abnormal meiosis when the influences from activated WNT/β-catenin signaling exceed a threshold. Functions of other miRNAs in meiosis in vivo have not been described well. miR-17-92 knockout mouse showed abnormal spermatogenesis with reduced number of sperm, though detailed mechanisms including its target mRNAs were not reported [79].

We also found that overexpression of *miR-871* and/or *miR-880* in GSCs repressed the increase in number, and *Fzd4* expression (Fig 8A–8D). In addition, stable β-catenin rescued decreased GSC number by *miR-871* or *miR-880* (Fig 8E and 8F and S7 Table). These results suggest that control of the expression levels of *Fzd4* and subsequent WNT/β-catenin signaling activity by miR-871-3p and miR-880-3p are critical for proliferation and/or survival of GSCs. Meanwhile, repression of GSCs by XmiRs was not completely rescued by stable β-catenin (Fig 8E and 8F), suggesting that XmiRs repress the expression of additional targets other than WNT/β-catenin signaling related genes to regulate GSCs. In addition, it is also likely that XmiRs repress additional WNT/β-catenin signaling related genes other than *Fzd4*. According to this prediction, we found that *Dixdc1* encoding Disheveled-2-associated protein [80] and *Tbl1xr1* encoding β-catenin-associated protein [81] are potential targets of XmiRs (S6 Table).

Previous studies showed functions of miRNAs in SSCs. For instance, proliferation of SSCs is stimulated by miR-20 and miR-106 via repressing the expression of *Ccnd1* (Cyclin D1) and *Stat3* (signal transducer and activator of transcription 3) [18], and by miR-224 via repressing *Dmrt1* (Doublesex and mab-3 related transcription factor 1) [19], while survival of SSCs is supported by miR-146 via repressing *Med1* (Mediator of RNA polymerase II transcription subunit 1) encoding a retinoic acid receptor associating protein [20]. In addition, miR-202 [24] and miR-221/222 [25] maintain SSCs in undifferentiated status via repressing *Rbfox2* (RNA binding fox-1 homolog 2) and unknown direct targets, respectively. We found that XmiRs repressed GSCs to increase in number in culture, and repression of a WNT receptor, FZD4 and of possible additional targets by XmiR may be involved. The results together suggest that SSCs are maintained by the functions of miRNAs via various molecular pathways.

In the normal context, appropriate levels of WNT/β-catenin signaling molecules are crucial for spermatogonia and spermatocytes, and miR871-3p and miR-880-3p in those cells may contribute to fine tuning of the levels of WNT/β-catenin signaling activity via regulation of *Fzd4* expression. The expression of Fzd4 mRNA was decreased at zygotene-pachytene transition in meiosis (Fig 7C), while FZD4 protein expression was upregulated in pachytene/diplotene spermatocytes and round spermatids (S6 Fig). With regard to the expression of XmiRs, miR-880-3p and miR-871-3p was downregulated in preleptotene and pachytene/diplotene spermatocytes, respectively (Fig 7D and 7E). The results suggest that miR-880-3p is involved in de-stabilization of Fzd4 mRNA, while miR-871-3p may repress translation of Fzd4 mRNA. It is likely that Fzd4 mRNA starts to translate immediately after downregulation of miR-871-3p at zygotene-pachytene transition, and FZD4 protein may stably persist in pachytene spermatocytes onwards. Identification of downstream molecules of WNT/β-catenin signaling that directly function in spermatogenesis is an important subject for future studies.

## Supporting information

S1 Fig Testis weight and diameter of seminiferous tubules of XmiR-deficient mouse. (A, C) Testis weight / body weight of *ΔmiR-741* (OT16; n = 2), *ΔmiR-871* (OT17; n = 2) and *ΔmiR-880* (OT49; n = 2) (A), of *ΔXmiRs* (OT84; n = 6) (B), and of their wildtype littermates (n = 6 for A and n = 6 for C) at 8 weeks of age for A and 12 weeks of age for C. (B, D) Diameter of seminiferous tubules of *ΔmiR-741* (OT16; n = 1), *ΔmiR-871* (OT17; n = 1) and *ΔmiR-880* (OT49; n = 1) (B), of *ΔXmiRs* (OT84; n = 3) (D), and of their wildtype littermates (n = 1 for B and n = 3 for D) at 8 weeks of age for B and 12 weeks of age for D. Fifteen seminiferous tubules in each section and three sections of each mouse were measured. *P < 0.05 and **P < 0.01.
(TIF)

S2 Fig Localization of γH2AX in spermatocytes in *ΔXmiR* testes. Testis sections were co-stained by anti-SCP3 (red) and anti-γH2AX (cyan) antibodies in WT and *ΔXmiRs* (F2 generation of OT100) mice. Arrowheads show spermatocytes of the indicated stages. The fourth column shows higher magnification views corresponding to the rectangular area in the pictures in the third columns. Scale bars = 50 μm (the third columns), 25 μm (the first, second and fourth columns).
(TIF)

S3 Fig Venn diagram showing the relationship of putative target mRNAs of miR-871-3p and miR-880-3p. Corresponding gene lists are shown in S6 Table.
(TIF)

S4 Fig The expression of the putative common target genes of miR-871-3p and miR-880-3p in the testes of WT and *ΔXmiRs* mice. Relative expression of the putative common target genes of miR-871-3p and miR-880-3p in the testes of WT and *ΔXmiRs* (F2 of the OT84 line) mice at 12 weeks of age was determined by quantitative RT-PCR analysis. The expression in WT testis was set as 1. Error bars represent standard errors of three biological replicates.
(TIF)

S5 Fig The expression of β-catenin in *ΔXmiR* testes. (A) Sections of WT and *ΔXmiR* (OT84) testes at 12 weeks of age were co-stained by anti- β-catenin (red) and anti-Plzf (cyan) antibodies. The second and fourth column show higher magnification views corresponding to the rectangular area in the pictures in the first and third columns. Arrows and arrowheads show Plzf-positive SSCs with intense and faint fluorescence, respectively, for β-catenin. Scale bars = 50 μm (the first, the third columns), 25 μm (second and fourth columns). (B) Quantitative estimation of the expression of β-catenin protein in Plzf-positive SSCs in WT and *ΔXmiR* testes. Relative signal intensity in nucleus and cytoplasm of SSCs compared with that in Leydig cells is shown. Four and eleven Plzf-positive cells in a single *ΔXmiR* and WT mouse, respectively, were measured. **P < 0.01.
(TIF)

S6 Fig The expression of FZD4 in WT testes. Testis sections were co-stained by anti-SCP3 (red) and anti-FZD4 (green) antibodies in WT. The second and fourth column show higher magnification views corresponding to the rectangular area in the pictures in the first and third columns. White arrowheads: leptotene spermatocytes, yellow arrowheads: zygotene spermatocytes, white arrows: pachytene spermatocytes, yellow arrows: diplotene spermatocytes. Scale bars = 50 μm (the first, the third columns), 25 μm (second and fourth columns).
(TIF)

S7 Fig A heat map of miRNAs highly expressed in testis or spermatogonia. Relative miRNA expression is described according to the color scale. Red and green indicate high and low expression, respectively. Mouse embryonic fibroblasts (MEFs), embryonic stem (ES) cells, primordial germ cells (PGCs), spermatogonia (SPG), spermatozoa (SPZ).
(TIF)

S1 Table Small RNA-seq data used for this study. ES: embryonic stem cells, MEFs: mouse embryonic fibroblasts, PGCs: primordial germ cells.
(DOCX)

S2 Table Top 20 miRNAs highly expressed in PGCs (corresponding to Fig 1B). Read counts of each miRNA normalized to reads per million (RPM) were shown. miR-741-3p, miR-871-3p, and miR-880-3p were highlighted by yellow. ES: embryonic stem cell, mouse embryonic fibroblasts (MEFs), PGCs: primordial germ cells, SPG: spermatogonia, SPZ: spermatozoa.
(DOCX)

S3 Table Lists of predicted target genes of miR-741-3p, miR-871-3p, and miR-880-3p (corresponding to Fig 4B). (XLSX)

S4 Table Fertility of *ΔXmiRs* mice. Three hemizygous *ΔXmiRs* F2 males of the OT100 line (#2, 4, 5) and their WT littermates (#1, 3, 6) were mated twice each with MCH females. Three homozygous *ΔXmiRs* F2 females of the OT84 line (#2, 3, 6) and their heterozygous littermates (#4, 10, 11) were mated once with Oct4-*ΔPE-GFP* transgenic males. The number of pups is shown.
(DOCX)

S5 Table Ratios of abnormal seminiferous tubules. Ratios of abnormal seminiferous tubules (% of abnormal seminiferous tubules in total seminiferous tubules) in *ΔXmiRs* mice at 8, 12, 16, and 30 weeks of age. Abnormal seminiferous tubules were counted in three sections from each mouse. Testis sections were prepared from three WT mice and one mouse of each *ΔXmiRs* line (OT84, OT97, and OT100). ND: not determined.
(DOCX)

S6 Table Lists of putative target mRNAs of miR-871-3p and miR-880-3p (corresponding to S3 Fig). (XLSX)

S7 Table Relative number of GSCs with the expression vector of stable β-catenin compared with those with control expression vector. Values for GSCs with miR-871, miR-880 and control miR expression vectors based on the data in Fig 8F are shown.
(DOCX)

S8 Table miRNAs that are predicted to target the *Fzd4* 3’-UTR expressed in germ cells (corresponding to S7 Fig). Read counts of each miRNA normalized to reads per million (RPM) were shown. ES: embryonic stem cell, mouse embryonic fibroblasts (MEFs), PGCs: primordial germ cells, SPG: spermatogonia, SPZ: spermatozoa.
(DOCX)

S9 Table List of primers used in this study. (DOCX)
