# Supplementary figures and images for: Identification of the X-linked germ cell specific miRNAs (XmiRs) and their functions
Source: PLoS One. 2019 Feb 1;14(2):e0211739. doi: 10.1371/journal.pone.0211739 (PMC6358104; doi:10.1371/journal.pone.0211739)

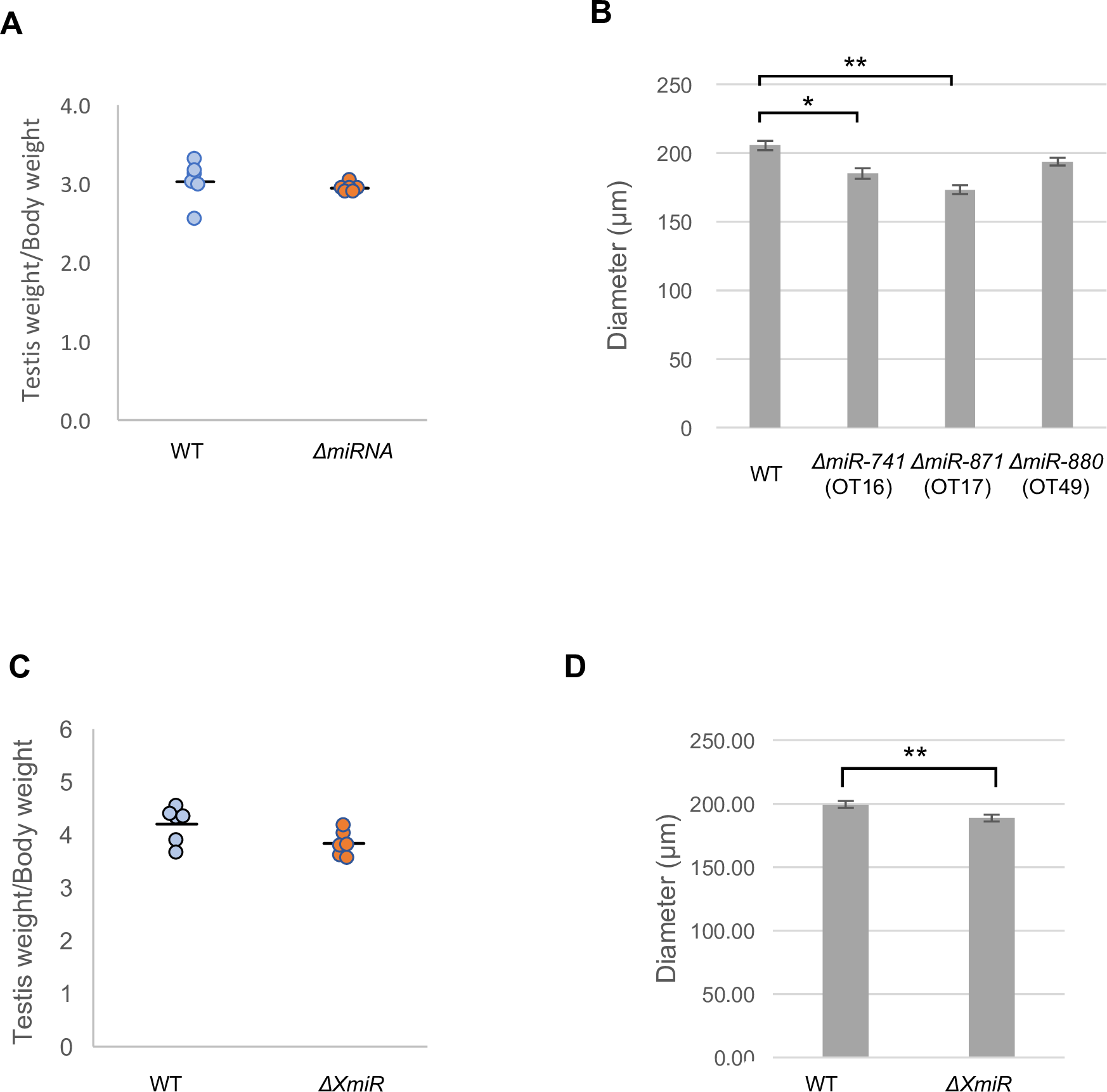

Supplement: S1 Fig — (A, C) Testis weight / body weight of ΔmiR-741 (OT16; n = 2), ΔmiR-871 (OT17; n = 2) and ΔmiR-880 (OT49; n = 2) (A), of ΔXmiRs (OT84; n = 6) (B), and of their wildtype littermates (n = 6 for A and n = 6 for C) at 8 weeks of age for A and 12 weeks of age for C. (B, D) Diameter of seminiferous tubules of ΔmiR-741 (OT16; n = 1), ΔmiR-871 (OT17; n = 1) and ΔmiR-880 (OT49; n = 1) (B), of ΔXmiRs (OT84; n = 3) (D), and of their wildtype littermates (n = 1 for B and n = 3 for D) at 8 weeks of age for B and 12 weeks of age for D. Fifteen seminiferous tubules in each section and three sections of each mouse were measured. *P < 0.05 and **P < 0.01. (TIF) [file pone.0211739.s001.tif]

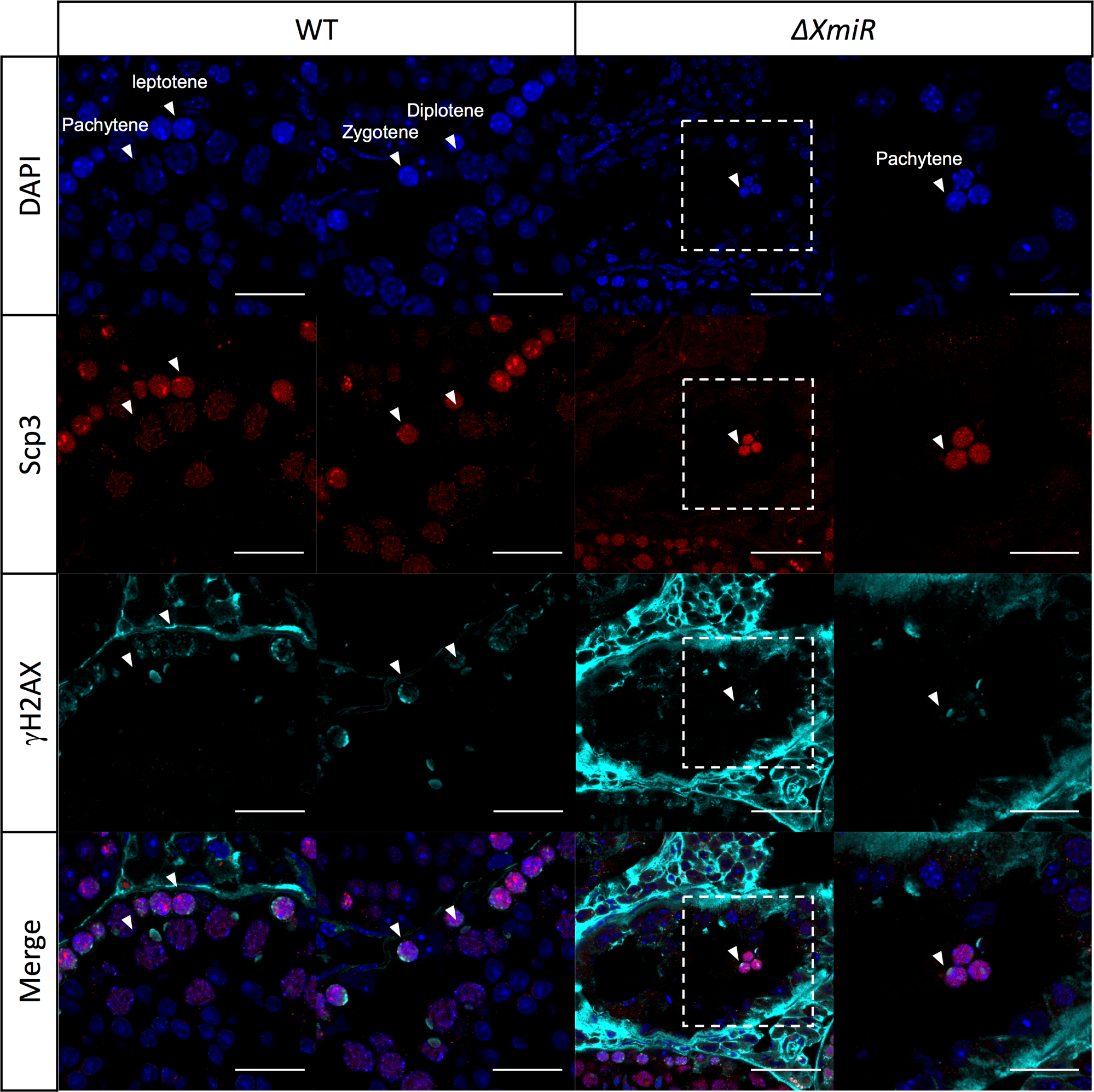

Supplement: S2 Fig — Testis sections were co-stained by anti-SCP3 (red) and anti-γH2AX (cyan) antibodies in WT and ΔXmiRs (F2 generation of OT100) mice. Arrowheads show spermatocytes of the indicated stages. The fourth column shows higher magnification views corresponding to the rectangular area in the pictures in the third columns. Scale bars = 50 μm (the third columns), 25 μm (the first, second and fourth columns). (TIF) [file pone.0211739.s002.tif]

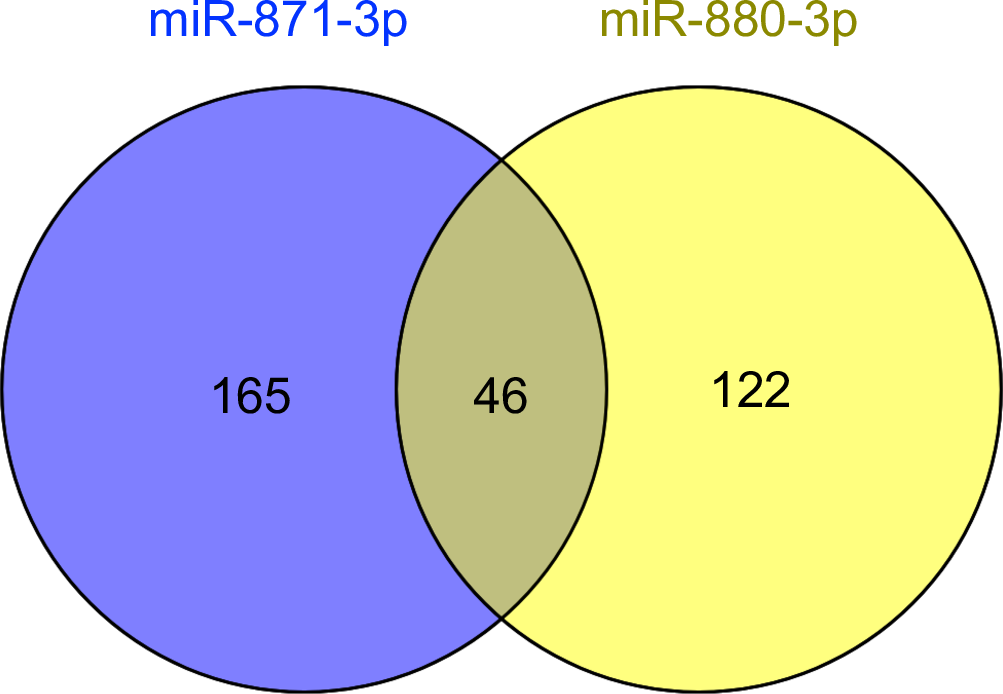

Supplement: S3 Fig — Corresponding gene lists are shown in S6 Table. (TIF) [file pone.0211739.s003.tif]

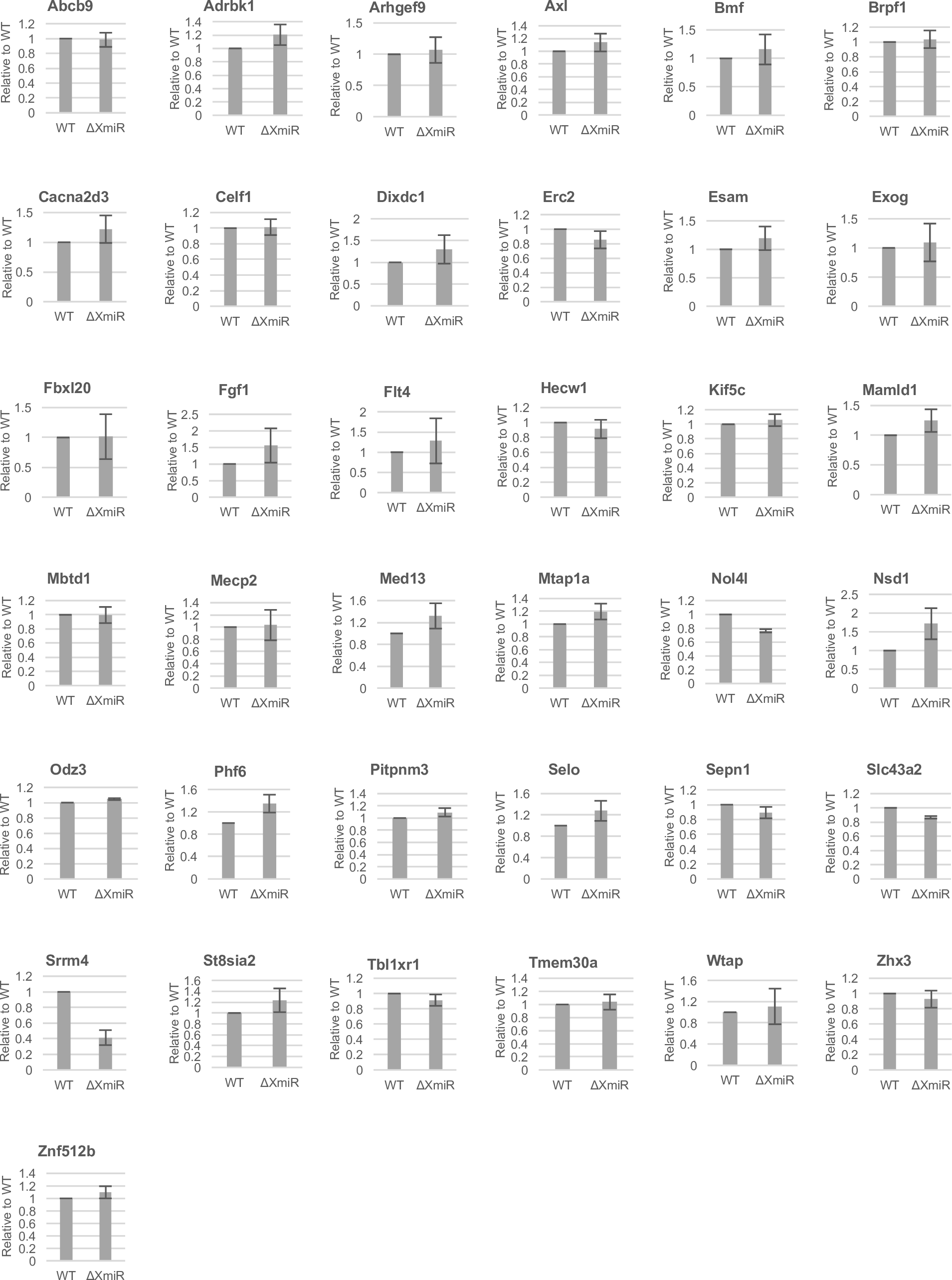

Supplement: S4 Fig — Relative expression of the putative common target genes of miR-871-3p and miR-880-3p in the testes of WT and ΔXmiRs (F2 of the OT84 line) mice at 12 weeks of age was determined by quantitative RT-PCR analysis. The expression in WT testis was set as 1. Error bars represent standard errors of three biological replicates. (TIF) [file pone.0211739.s004.tif]

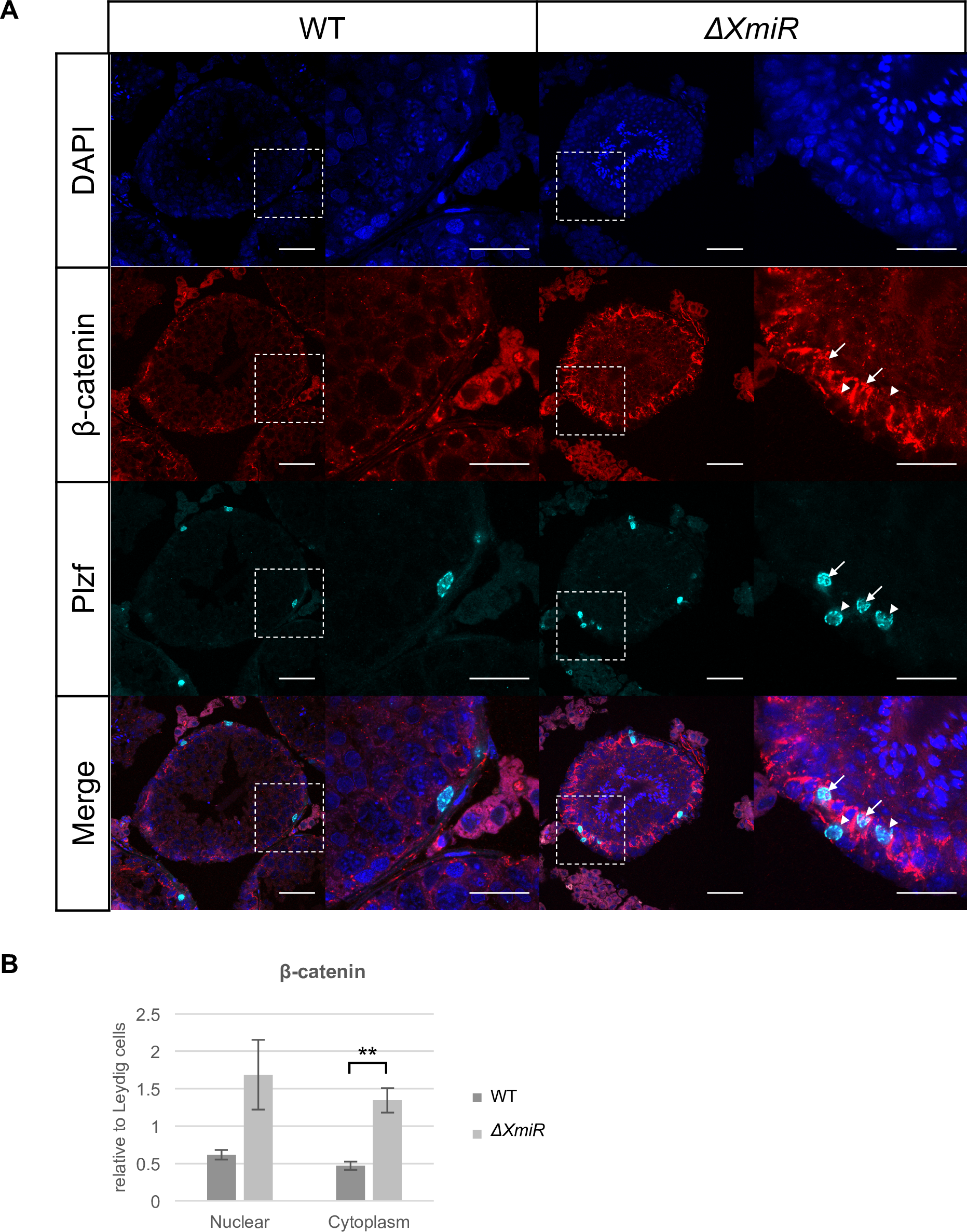

Supplement: S5 Fig — (A) Sections of WT and ΔXmiR (OT84) testes at 12 weeks of age were co-stained by anti- β-catenin (red) and anti-Plzf (cyan) antibodies. The second and fourth column show higher magnification views corresponding to the rectangular area in the pictures in the first and third columns. Arrows and arrowheads show Plzf-positive SSCs with intense and faint fluorescence, respectively, for β-catenin. Scale bars = 50 μm (the first, the third columns), 25 μm (second and fourth columns). (B) Quantitative estimation of the expression of β-catenin protein in Plzf-positive SSCs in WT and ΔXmiR testes. Relative signal intensity in nucleus and cytoplasm of SSCs compared with that in Leydig cells is shown. Four and eleven Plzf-positive cells in a single ΔXmiR and WT mouse, respectively, were measured. **P < 0.01. (TIF) [file pone.0211739.s005.tif]

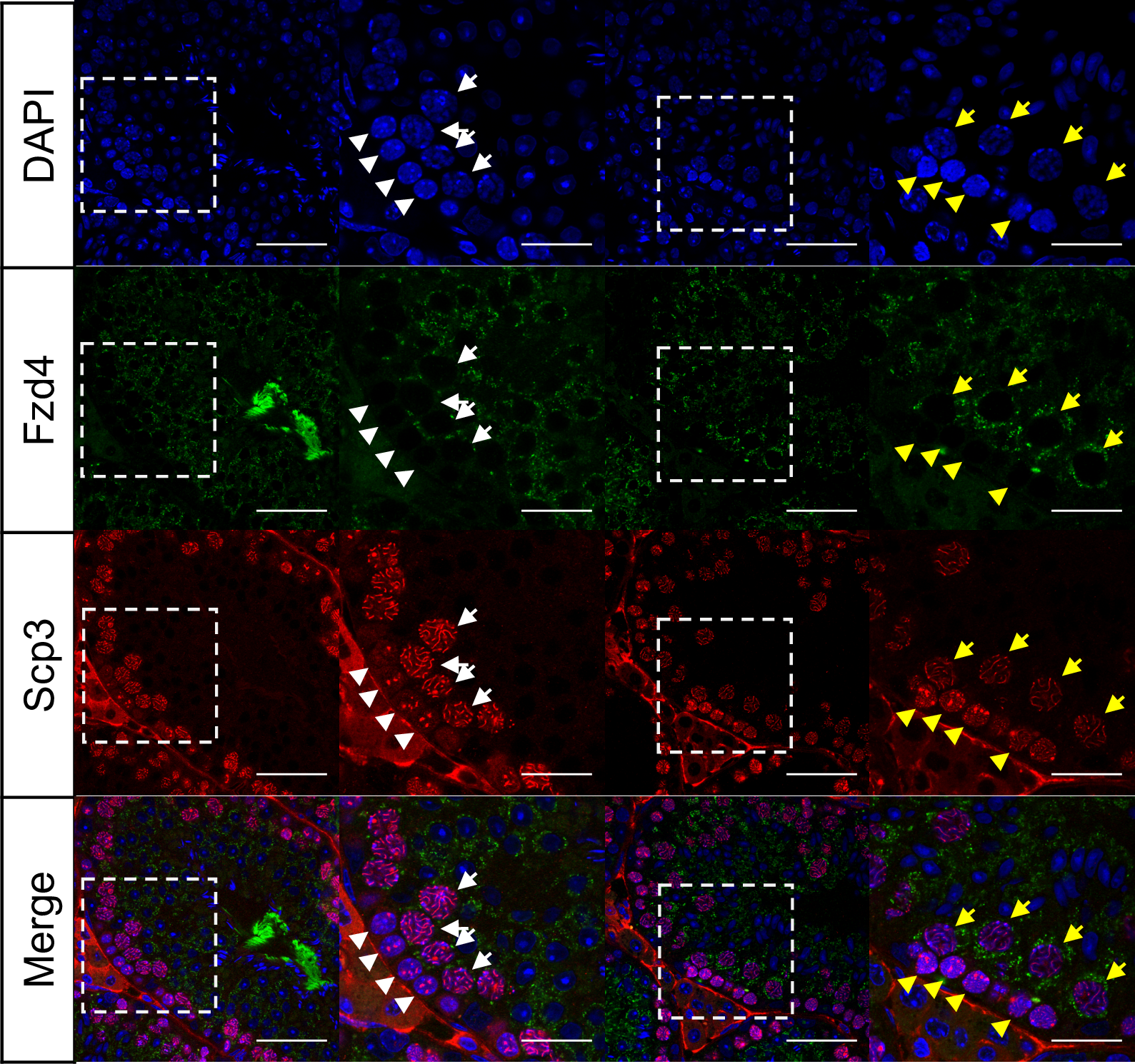

Supplement: S6 Fig — Testis sections were co-stained by anti-SCP3 (red) and anti-FZD4 (green) antibodies in WT. The second and fourth column show higher magnification views corresponding to the rectangular area in the pictures in the first and third columns. White arrowheads: leptotene spermatocytes, yellow arrowheads: zygotene spermatocytes, white arrows: pachytene spermatocytes, yellow arrows: diplotene spermatocytes. Scale bars = 50 μm (the first, the third columns), 25 μm (second and fourth columns). (TIF) [file pone.0211739.s006.tif]

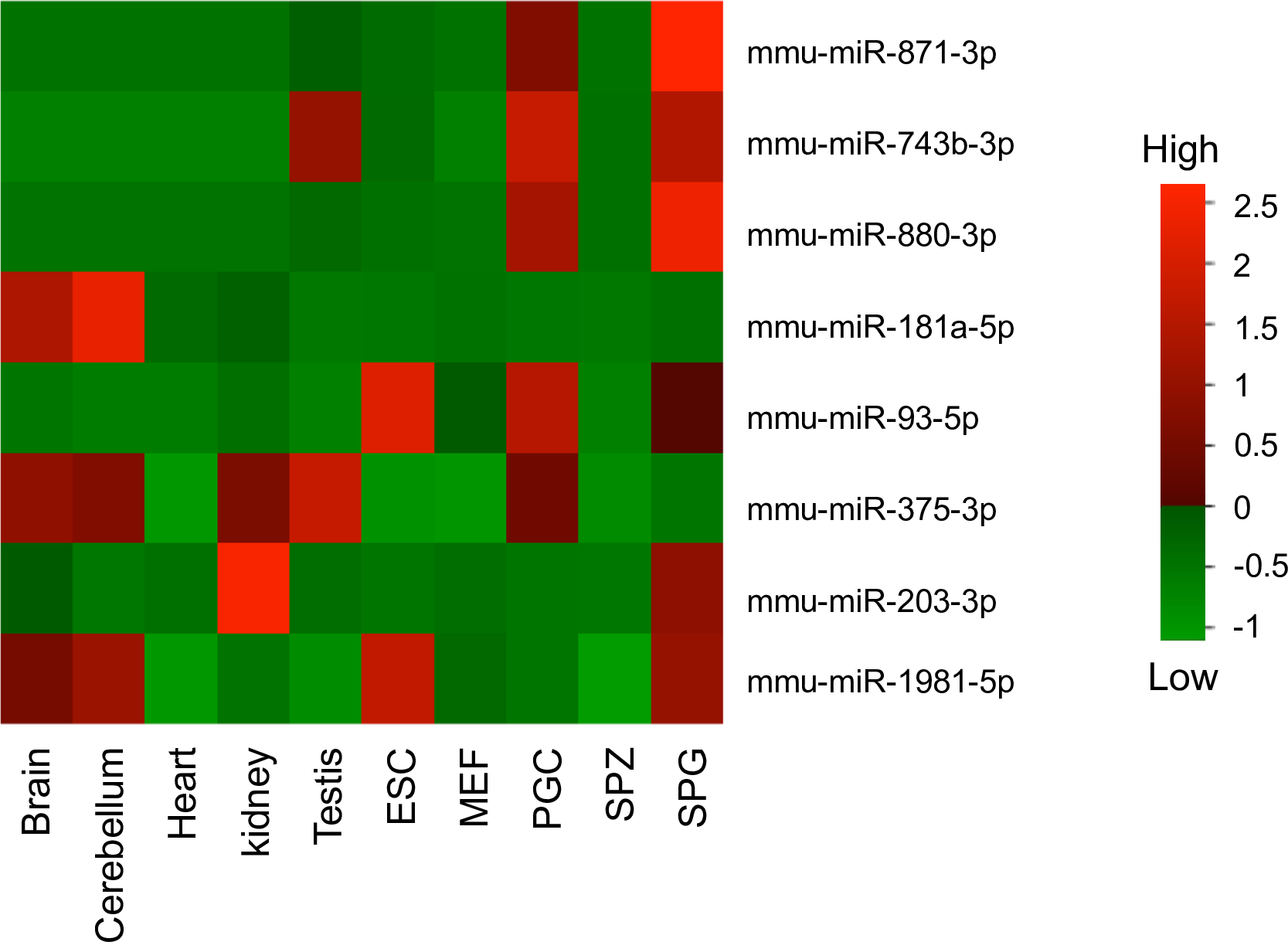

Supplement: S7 Fig — Relative miRNA expression is described according to the color scale. Red and green indicate high and low expression, respectively. Mouse embryonic fibroblasts (MEFs), embryonic stem (ES) cells, primordial germ cells (PGCs), spermatogonia (SPG), spermatozoa (SPZ). (TIF) [file pone.0211739.s007.tif]
