# Supplementary material for: Identification of the X-linked germ cell specific miRNAs (XmiRs) and their functions
Source: PLoS One. 2019 Feb 1;14(2):e0211739. doi: 10.1371/journal.pone.0211739 (PMC6358104; doi:10.1371/journal.pone.0211739)
Supplement: S1 Table — ES: embryonic stem cells, MEFs: mouse embryonic fibroblasts, PGCs: primordial germ cells. (DOCX) [file pone.0211739.s008.docx]

**S1 Table.**

| Run | Tissue | Total reads | aligned genome | miRNA | Reference |
| --- | --- | --- | --- | --- | --- |
| SRR553582 | Brain | 23,144,542 | 20,107,924  (86.88 %) | 14,850,742  (64.17 %) | GSE40499  (Meunier. et al. 2013) |
| SRR553583 | Cerebellum | 18,944,090 | 16,616,439  (87.71 %) | 12,873,742  (67.96 %) |  |
| SRR553584 | Heart | 32,807,210 | 29,675,341  (90.45 %) | 21,643,367  (65.97 %) |  |
| SRR553585 | Kidney | 19,134,369 | 17,553,774  (91.74 %) | 9,926,408  (51.88 %) |  |
| SRR553586 | Testis | 25,924,146 | 23,345,305  (90.05 %) | 1,787,152  (6.89 %) |  |
| SRR1042095 | ES2 | 9,447,530 | 7,118,383  (75.35 %) | 3,636,910  (38.5 %) | GSE52950  (Zhao B. et al. 2014) |
| SRR1042096 | ES3 | 10,530,511 | 8,047,213  (76.42 %) | 4,321,115  (41.03 %) |  |
| SRR1042097 | ES4 | 10,127,396 | 7,592,011  (74.97 %) | 3,785,247  (37.38 %) |  |
| SRR1042098 | MEF13 | 10,929,710 | 10,334,888  (94.56 %) | 5,777,631  (52.86 %) |  |
| SRR1042099 | MEF14 | 10,397,097 | 9,699,108  (93.29 %) | 5,277,777  (50.76 %) |  |
| SRR1509751 | PGC | 42,643,955 | 38,302,296  (84.82 %) | 7,783,482  (18.25 %) | GSE59254  (García-López J. et al. 2015) |
| SRR1509750 | Spermatogonia | 42,438,528 | 37,296,548  (87.88 %) | 5,930,530  (13.97 %) |  |
| SRR1509748 | Spermatozoa | 28,486,445 | 18,956,126  (66.54 %) | 907,521  (3.19 %) |  |
