# Supplementary material for: Identification of the X-linked germ cell specific miRNAs (XmiRs) and their functions
Source: PLoS One. 2019 Feb 1;14(2):e0211739. doi: 10.1371/journal.pone.0211739 (PMC6358104; doi:10.1371/journal.pone.0211739)
Supplement: S2 Table — Read counts of each miRNA normalized to reads per million (RPM) were shown. miR-741-3p, miR-871-3p, and miR-880-3p were highlighted by yellow. ES: embryonic stem cell, mouse embryonic fibroblasts (MEFs), PGCs: primordial germ cells, SPG: spermatogonia, SPZ: spermatozoa. (DOCX) [file pone.0211739.s009.docx]

|  | Brain | Cerebellum | Heart | kidney | Testis | ESC | MEF | PGC | SPZ | SPG |
| --- | --- | --- | --- | --- | --- | --- | --- | --- | --- | --- |
| mmu-miR-182-5p | 14974.15 | 383.60 | 251.66 | 1208.97 | 47.12 | 78374.82 | 873.57 | 73449.23 | 148.08 | 18051.74 |
| mmu-miR-183-5p | 6873.91 | 110.37 | 138.36 | 239.04 | 10.15 | 30814.03 | 365.82 | 15288.27 | 38.02 | 2404.50 |
| mmu-miR-148a-3p | 863.09 | 38.70 | 1314.46 | 467.88 | 364.27 | 6505.92 | 5315.32 | 10099.92 | 647.27 | 15548.80 |
| mmu-miR-93-5p | 931.72 | 412.30 | 408.29 | 1347.74 | 133.35 | 12224.28 | 2755.56 | 9537.60 | 178.11 | 3128.33 |
| mmu-miR-103-3p | 9374.26 | 4031.73 | 622.74 | 2381.65 | 436.06 | 11167.77 | 6528.68 | 7348.62 | 96.36 | 7414.07 |
| mmu-miR-871-3p | 0.25 | 2.17 | 0.84 | 0.11 | 1406.92 | 426.85 | 0.45 | 5330.75 | 7.24 | 14410.49 |
| mmu-miR-880-3p | 0.10 | 0.36 | 0.30 | 0.11 | 388.26 | 81.10 | 0.11 | 4587.66 | 82.82 | 7618.01 |
| mmu-miR-20a-5p | 118.46 | 6.50 | 77.24 | 430.90 | 66.99 | 6270.42 | 1869.12 | 4385.66 | 72.79 | 1018.25 |
| mmu-miR-295-3p | 0.00 | 0.06 | 0.00 | 0.00 | 0.30 | 15265.38 | 1.07 | 3642.29 | 0.78 | 217.71 |
| mmu-miR-99b-5p | 9865.31 | 46778.80 | 3498.46 | 8331.09 | 177.89 | 8075.85 | 14619.03 | 3579.08 | 19.47 | 10192.17 |
| mmu-miR-291a-3p | 0.00 | 0.00 | 0.00 | 0.00 | 0.00 | 11767.36 | 0.78 | 3188.22 | 0.13 | 417.17 |
| mmu-miR-24-3p | 7016.04 | 2381.62 | 1176.46 | 5836.86 | 120.02 | 6600.35 | 7686.54 | 3131.82 | 51.88 | 5705.75 |
| mmu-miR-30e-3p | 1283.87 | 376.68 | 1667.68 | 1450.51 | 137.03 | 1113.82 | 514.64 | 2651.30 | 22.44 | 2251.51 |
| mmu-miR-741-3p | 0.40 | 0.18 | 0.24 | 0.00 | 1973.42 | 314.80 | 0.55 | 2519.67 | 236.78 | 4776.98 |
| mmu-miR-17-5p | 74.75 | 17.87 | 33.66 | 240.80 | 38.12 | 3947.71 | 1289.81 | 2182.09 | 24.91 | 638.32 |
| mmu-miR-872-5p | 1762.19 | 29.43 | 170.55 | 990.61 | 113.47 | 2781.71 | 1354.07 | 2040.53 | 30.83 | 780.43 |
| mmu-miR-148b-3p | 686.94 | 171.70 | 169.77 | 348.53 | 42.45 | 2012.19 | 627.25 | 1898.45 | 65.42 | 639.11 |
| mmu-miR-20b-5p | 4.53 | 1.75 | 0.74 | 84.37 | 0.09 | 2946.69 | 6.17 | 1880.88 | 0.13 | 52.38 |
| mmu-miR-96-5p | 378.56 | 0.84 | 6.77 | 19.20 | 0.26 | 1391.29 | 19.33 | 1824.22 | 15.12 | 681.26 |
| mmu-miR-27b-3p | 4138.07 | 677.28 | 1789.40 | 5770.55 | 35.38 | 3723.74 | 4931.39 | 1444.20 | 37.22 | 1677.08 |

**S2 Table.**
