# Supplementary material for: Identification of the X-linked germ cell specific miRNAs (XmiRs) and their functions
Source: PLoS One. 2019 Feb 1;14(2):e0211739. doi: 10.1371/journal.pone.0211739 (PMC6358104; doi:10.1371/journal.pone.0211739)
Supplement: S4 Table — Three hemizygous ΔXmiRs F2 males of the OT100 line (#2, 4, 5) and their WT littermates (#1, 3, 6) were mated twice each with MCH females. Three homozygous ΔXmiRs F2 females of the OT84 line (#2, 3, 6) and their heterozygous littermates (#4, 10, 11) were mated once with Oct4-ΔPE-GFP transgenic males. The number of pups is shown. (DOCX) [file pone.0211739.s011.docx]

**S4 Table.**

| WT male | # of pups | *ΔXmiR* male | # of pups | Hetero female | # of pups | *ΔXmiR* female | # of pups |
| --- | --- | --- | --- | --- | --- | --- | --- |
| OT100TF2#1 | 13 | OT100TF2#2 | 11 | OT84TF2#4 | 12 | OT84TF2#2 | 8 |
| OT100TF2#1 | 12 | OT100TF2#2 | 8 | OT84TF2#10 | 10 | OT84TF2#3 | 9 |
| OT100TF2#3 | 13 | OT100TF2#4 | 9 | OT84TF2#11 | 11 | OT84TF2#6 | 11 |
| OT100TF2#3 | 13 | OT100TF2#4 | 14 |  |  |  |  |
| OT100TF2#6 | 13 | OT100TF2#5 | 13 |  |  |  |  |
| OT100TF2#6 | 11 | OT100TF2#5 | 13 |  |  |  |  |
| Average | 12.50 |  | 11.33 |  | 11 |  | 9.33 |
| SE | 0.34 |  | 0.99 |  | 0.58 |  | 0.88 |
| p-value |  |  | 0.29 |  |  |  | 0.19 |
