# Supplementary material for: Identification of the X-linked germ cell specific miRNAs (XmiRs) and their functions
Source: PLoS One. 2019 Feb 1;14(2):e0211739. doi: 10.1371/journal.pone.0211739 (PMC6358104; doi:10.1371/journal.pone.0211739)
Supplement: S5 Table — Ratios of abnormal seminiferous tubules (% of abnormal seminiferous tubules in total seminiferous tubules) in ΔXmiRs mice at 8, 12, 16, and 30 weeks of age. Abnormal seminiferous tubules were counted in three sections from each mouse. Testis sections were prepared from three WT mice and one mouse of each ΔXmiRs line (OT84, OT97, and OT100). ND: not determined. (DOCX) [file pone.0211739.s012.docx]

**S5 Table.**

|  | WT | OT84 | OT87 | OT100 |
| --- | --- | --- | --- | --- |
| 8 weeks | 0.0 | 0.0 | 0.9 | 1.7 |
| 12 weeks | 0.0 | 1.1 | 1.4 | 2.9 |
| 16 weeks | 0.0 | 1.3 | 2.1 | 3.1 |
| 30 weeks | 0.0 | 4.2 | ND | ND |
