# Supplementary material for: Identification of the X-linked germ cell specific miRNAs (XmiRs) and their functions
Source: PLoS One. 2019 Feb 1;14(2):e0211739. doi: 10.1371/journal.pone.0211739 (PMC6358104; doi:10.1371/journal.pone.0211739)
Supplement: S7 Table — Values for GSCs with miR-871, miR-880 and control miR expression vectors based on the data in Fig 8F are shown. (DOCX) [file pone.0211739.s014.docx]

S7 Table

|  | Control | *miR-871* | *miR-880* |
| --- | --- | --- | --- |
| Relative value of Day 10 (Control to β-Catenin) | 1.47 | 3.24 | 3.26 |
| SE | 0.03 | 0.02 | 0.25 |
| p-value |  | 0.0000 | 0.0021 |
