# Supplementary material for: Identification of the X-linked germ cell specific miRNAs (XmiRs) and their functions
Source: PLoS One. 2019 Feb 1;14(2):e0211739. doi: 10.1371/journal.pone.0211739 (PMC6358104; doi:10.1371/journal.pone.0211739)
Supplement: S8 Table — Read counts of each miRNA normalized to reads per million (RPM) were shown. ES: embryonic stem cell, mouse embryonic fibroblasts (MEFs), PGCs: primordial germ cells, SPG: spermatogonia, SPZ: spermatozoa. (DOCX) [file pone.0211739.s015.docx]

| Mirna Name | Brain | Cerebellum | Heart | kidney | Testis | Es cell | MEF | PGC | SPZ | SPG |
| --- | --- | --- | --- | --- | --- | --- | --- | --- | --- | --- |
| mmu-miR-871-3p | 0.25 | 2.17 | 0.84 | 0.11 | 1406.92 | 426.85 | 0.45 | 5330.75 | 7.24 | 14410.49 |
| mmu-miR-743b-3p | 0 | 0 | 0 | 0.06 | 292.09 | 59.45 | 0.05 | 417.02 | 40.41 | 364 |
| mmu-miR-880-3p | 0.1 | 0.36 | 0.3 | 0.11 | 388.26 | 81.1 | 0.11 | 4587.66 | 82.82 | 7618.01 |
| mmu-miR-181a-5p | 26114.23 | 37951.21 | 2987.7 | 4819.65 | 100.02 | 229.8 | 1289.94 | 113.26 | 4.48 | 1903.45 |
| mmu-miR-93-5p | 931.72 | 412.3 | 408.29 | 1347.74 | 133.35 | 12224.28 | 2755.56 | 9537.6 | 178.11 | 3128.33 |
| mmu-miR-375-3p | 87.83 | 77.45 | 1.95 | 74 | 124.35 | 5.8 | 3.48 | 66.71 | 10.08 | 24.32 |
| mmu-miR-203-3p | 257.46 | 11.86 | 61.53 | 1772.38 | 102.42 | 24.74 | 97.46 | 17.15 | 12.25 | 843 |
| mmu-miR-1981-5p | 719.52 | 974.1 | 29.86 | 273.79 | 107.82 | 1230.11 | 346.41 | 264.48 | 1.72 | 942.86 |

S8 Table
