# Supplementary material for: Identification of the X-linked germ cell specific miRNAs (XmiRs) and their functions
Source: PLoS One. 2019 Feb 1;14(2):e0211739. doi: 10.1371/journal.pone.0211739 (PMC6358104; doi:10.1371/journal.pone.0211739)
Supplement: S9 Table — (DOCX) [file pone.0211739.s016.docx]

**S9 Table.**

| gRNA expression vector construction | |
| --- | --- |
| miR-741-3p-1 sense | TAGGGCTTACATGATTTACATGAT |
| miR-741-3p-1 antisense | AAACATCATGTAAATCATGTAAGC |
| miR-871-3p-1 sense | TAGGACATATGACTGGCACCATTC |
| miR-871-3p-1 antisense | AAACGAATGGTGCCAGTCATATGT |
| miR-880-3p-1 sense | TAGGCATCCTCTCTGAGTAGAGTA |
| miR-880-3p-1 antisense | AAACTACTCTACTCAGAGAGGATG |
| Semi-quantitative RT-PCR | |
| oligo(dT)-RACE primer | CTAATACGACTCACTATAGGGCGACCACGCGTATCGATGTCGACTTTTTTTTTTTTTTTTV |
| RACE PCR Universal primer | CTAATACGACTCACTATAGGGC |
| RNU6B Fw | CACGCAAATTCGTGAAGCGTTCC |
| miR741-3p Fw | TGAGAGATGCCATTCTATGTAGA |
| miR871-3p Fw | TGACTGGCACCATTCTGGATAAT |
| miR880-3p Fw | TACTCCATCCTCTCTGAGTAGA |
| Quantitative RT-PCR | |
| Chdh qPCR Fw | CAGGCCCTTAGAGGCTGGA |
| Chdh qPCR Rev | CAAAGGTGTACTCATCCTTGCC |
| Fzd4 qPCR Fw | TGCCAGAACCTCGGCTACA |
| Fzd4 qPCR Rev | ATGAGCGGCGTGAAAGTTGT |
| Magix qPCR Rev | CAGAATCCAGACCACTAACACC |
| Magix qPCR Fw | AACACGGCAGACCCTAGAG |
| Mllt3 qPCR Fw | CGTCTTCCACTTGCACGAAAG |
| Mllt3 qPCR Rev | CCCGGACTCTTCTACCTTGTAA |
| Stox2 qPCR Fw | ATGTCCCCCATCAGTCAGTCT |
| Stox2 qPCR Rev | GTACCAGTGTGTTTAGGGTATGC |
| Pitx2 qPCR Fw | GCAGCCGTTGAATGTCTCTTC |
| Pitx2 qPCR Rev | GTCCGTGAACTCGACCTTTTT |
| Jun qPCR Fw | CCTTCTACGACGATGCCCTC |
| Jun qPCR Rev | GGTTCAAGGTCATGCTCTGTTT |
| Axin2 qPCR Fw | TGACTCTCCTTCCAGATCCCA |
| Axin2 qPCR Rev | TGCCCACACTAGGCTGACA |
| Sox17 qPCR Fw | GATGCGGGATACGCCAGTG |
| Sox17 qPCR Rev | CCACCACCTCGCCTTTCAC |
| cMyc qPCR Fw | AGCCCCTAGTGCTGCATGA |
| cMyc qPCR Rev | TCCACAGACACCACATCAATTTC |
| Luciferase reporter assay vector construction | |
| Fzd4_3'UTR_Xho I Fw | CCGCTCGAGCGGCTTCCTCGTTCCTCATTG |
| Fzd4_3'UTR_Not I Rev | ATAAGAATGCGGCCGCCAGGTTGCCTTCTGGTTTCTGG |
| Fzd4_927_Not I_Rev | GAAGAACGCGGCCGCGTTCTTCCAGACTGGCGTCT |
| Fzd4_927_Xho I_Fw | TCTCTCGAGAGACGCCAGTCTGGAAGAAC |
| Fzd4_2263_Not I_Rev | GCTGTGCGCGGCCGCGCACAGCAAAGCTAGTCAGCT |
| Fzd4 3’UTR delta-miR-871 Fw | CTGGAAGAACTGAAATGTTAAA |
| Fzd4 3’UTR delta-miR-871 Rev | TTTCAGTTCTTCCAGTCTGCCTAGATGCAATCACAC |
| Fzd4 3’UTR delta-miR-880-1 Fw | ATGTTGAGTCCCGCCTAGATTCCTGAAGC |
| Fzd4 3’UTR delta-miR-880-1 Rev | GGCGGGACTCAACATTTTGATTTTTCACAGC |
| Fzd4 3’UTR delta-miR-880-2 Fw | TTTATATGATGCCTAATGAACCTCC |
| Fzd4 3’UTR delta-miR-880-2 Rev | TAGGCATCATATAAAGGCCACACTGCCTCCAGT |
| Kozak-Ctnnb1-Fw-for-CS2-EF-MCS | CCGGTCTCGAGAATTGCCACCATGGCTACTCAAGCTGACC |
| Ctnnb1-Rev-for-CS2-EF-MCS | AGAGGATCCGCGGCCTTACAGGTCAGTATCAAACCAGG |
| Ctnnb1-AAAA-Fw | CCGCCACAGCTCCTGCCCTGAGTGGCAAGGGCA |
| Ctnnb1-AAAA-Rev | AGCATGGATTCCAGCATCCAAGTAAGACTGCTGCTG |
| Ctnnb1-AAAA-AS | CAGGAGCTGTGGCGGTGGCACCAGCATGGATTCCAGC |
